# Parameter-Efficient LoRA-GRL Adaptation for Cross-Center Classification of Benign and Malignant Lung Nodules on Heterogeneous Standard-Dose Chest CT: A Multi-Institutional Study from Palestine

**DOI:** 10.3390/jimaging12090461

**Published:** 2026-09-21

**Authors:** Radwan Qasrawi, Razan AbuGhoush, Ghada Issa, Suliman Thwib, Malak Amro, Rand Al Taweel, Sara Asfour, Yazan Dibas, Tawfiq Abukeshek, Marwan Qubja

**Affiliations:** 1 Department of Computer Science, Al-Quds University, Jerusalem P144, Palestine; gissa@staff.alquds.edu (G.I.); sthwib@staff.alquds.edu (S.T.); malak.amro@staff.alquds.edu (M.A.); 2 Department of Computer Engineering, Istinye University, 34010 Istanbul, Turkey; 3 The Center of Technology and Innovation, Al-Quds University, Jerusalem P144, Palestine; 4 Department of Medical Imaging, Al-Quds University, Jerusalem P144, Palestine; sara4asfour17@gmail.com; 5 Department of Radiology, Al-Makassed Charity Hospital, Jerusalem P.O. Box 19482, Palestine; 6 Faculty of Medicine, Al-Quds University, Jerusalem P144, Palestine

**Keywords:** lung nodule classification, parameter-efficient fine-tuning, low-rank adaptation, gradient reversal layer, domain adaptation, vision transformer

## Abstract

Automated CT lung nodule classification suffers from domain shift across centers. We propose LoRA-GRL, combining Low-Rank Adaptation (LoRA) with adversarial domain harmonization via a Gradient Reversal Layer. Rank-8 LoRA modules were inserted into all attention and feed-forward linear projections (48 sites) of a frozen ViT-S backbone; a domain discriminator encouraged center-invariant features across three centers. Normal vs. Benign and Normal vs. Malignant tasks used 264 patients with patient-level five-fold stratified cross-validation. LoRA-GRL achieved AUCs of 0.9735 and 0.9869, close to full fine-tuning (0.9747 and 0.9863). Differences from strongest baselines fell within overlapping 95% CIs under paired bootstrap, indicating comparable discrimination, not superiority. Efficiency is the main advantage: only 0.789 M trainable parameters, a 96.4% reduction from 21.865 M, and inference latency matched full fine-tuning after adapter merging. Grad-CAM showed nodule-localized predictions but also slice-selection failures. For benign classification, higher AUC than plain LoRA came with higher specificity but lower sensitivity. For malignant classification, sensitivity was 0.9412 (5.9 pp above full fine-tuning) and specificity 0.9697. The small cross-center AUC range (0.0011) reflects internal consistency across participating centers, not unseen-site generalization. LoRA-GRL is a promising parameter-efficient candidate for multi-center lung nodule classification, potentially reducing scanner-specific bias and storage/memory needs, but clinical utility requires external validation and prospective evaluation.

## 1. Introduction

Lung cancer remains the leading cause of cancer-related mortality worldwide, accounting for approximately 1.8 million deaths each year. Survival is strongly linked to the stage at diagnosis, with five-year survival rates exceeding 80% when the disease is detected early but dropping below 10% in patients with metastatic disease [1,2]. As a result, early identification of suspicious pulmonary nodules has become a central objective of lung cancer screening programs [3]. Large-scale screening studies have shown that low-dose computed tomography (CT) can reduce lung cancer mortality by nearly 20% among high-risk populations [4]. However, the growing volume of screening examinations places substantial demands on radiology services, creating a need for reliable computer-aided diagnosis (CAD) systems that can support nodule assessment and risk stratification [5].

Deep learning has transformed automated lung nodule analysis over the past decade. Early approaches relied on handcrafted radiomic features describing nodule shape, texture, and density characteristics [6,7]. These methods were later replaced by convolutional neural networks (CNNs), which demonstrated superior ability to learn discriminative imaging patterns directly from CT data [8,9]. More recently, Vision Transformers (ViTs) have emerged as a promising alternative. By leveraging self-attention mechanisms, ViTs can capture long-range spatial relationships and global morphological characteristics that are often associated with malignant growth patterns [10,11]. When pre-trained on large image repositories and adapted to medical imaging tasks, these models have achieved competitive performance across a range of diagnostic applications, including lung nodule classification [12,13].

Despite these advances, one of the most important barriers to clinical deployment remains insufficient generalizability across healthcare institutions. Models trained on data from a single center often perform substantially worse when applied to images acquired elsewhere [14,15]. This phenomenon, commonly referred to as domain shift, arises from differences in scanner manufacturers, acquisition parameters, reconstruction algorithms, slice thicknesses, and local imaging protocols. Although such variations may appear subtle to human observers, deep learning models can inadvertently learn scanner-specific patterns that are unrelated to the actual pathology. Consequently, a model that performs exceptionally well during internal validation may experience a notable decline in performance when evaluated on external data, limiting its practical utility in real-world screening environments [16].

Several studies have attempted to address this challenge through domain adaptation techniques. Among the most influential approaches is the Domain-Adversarial Neural Network (DANN), which uses a Gradient Reversal Layer (GRL) to encourage the learning of domain-invariant representations [17]. During training, the GRL forces the feature extractor to suppress information that distinguishes one institution from another while preserving features relevant to the clinical classification task. This strategy has improved robustness across heterogeneous datasets in multiple medical imaging applications, including histopathology [18], retinal imaging, and brain MRI analysis [19]. However, most existing domain adaptation frameworks rely on full-network fine-tuning, requiring millions of trainable parameters and substantial computational resources.

At the same time, the rapid growth of large pre-trained models has stimulated interest in parameter-efficient fine-tuning (PEFT) methods. Rather than updating an entire network, PEFT approaches adapt only a small subset of parameters while keeping the backbone largely frozen [20,21]. Low-Rank Adaptation (LoRA) [22] has emerged as one of the most effective strategies in this category. By representing weight updates through low-rank matrices, LoRA dramatically reduces the number of trainable parameters without increasing inference complexity. Several alternative parameter-efficient fine-tuning strategies have been proposed alongside LoRA. Adapter modules insert additional trainable bottleneck layers in series within each transformer block [23], which adds sequential computation and inference latency that scales with network depth. Prompt-tuning methods instead learn a set of continuous input tokens while leaving all backbone weights untouched [24]. Because they act only at the input stage, they have limited capacity to reshape the intermediate feature interactions distributed throughout the network, a property that may be needed to suppress scanner-specific artifacts. BitFit restricts training to the bias terms only [25] and, while extremely lightweight, offers markedly less representational flexibility than low-rank weight updates. LoRA, in contrast, injects additive low-rank updates directly into the attention projection matrices. Because these updates run parallel to the frozen weights rather than in series with them, they can be merged into the backbone after training, adding zero inference latency [22]. This combination of representational flexibility and negligible deployment overhead motivated the choice of LoRA as the adaptation mechanism paired with GRL in this study. Recent work by Veasey and Amini [26] demonstrated that LoRA-adapted Vision Transformers can achieve state-of-the-art performance in lung nodule classification while requiring substantially fewer trainable parameters than conventional fine-tuning. Nevertheless, these studies focused primarily on classification accuracy and did not explicitly address the challenge of cross-center domain variability.

This highlights an important gap in the current literature. Domain adaptation methods have traditionally focused on improving robustness across institutions, whereas parameter-efficient fine-tuning methods have focused on reducing computational cost. These two research directions have largely evolved independently, and it remains uncertain whether the limited representational capacity introduced by low-rank adaptation can simultaneously support both accurate disease classification and effective domain harmonization. The present study was designed to investigate this question. To our knowledge, the integration of parameter-efficient adaptation with adversarial domain alignment for multi-center lung nodule analysis has not been systematically investigated.

To address domain shift in multi-center lung nodule classification, we propose LoRA-GRL, a unified framework that combines Low-Rank Adaptation (LoRA) with adversarial domain harmonization. The framework integrates lightweight LoRA modules into a frozen Vision Transformer backbone and employs a Gradient Reversal Layer (GRL) to learn scanner-invariant feature representations across heterogeneous CT centers. Unlike previous studies that considered parameter-efficient fine-tuning and domain adaptation separately, LoRA-GRL jointly improves classification performance, cross-center robustness, and computational efficiency within a single architecture. The framework was evaluated using data from three clinical centers in Palestine, providing a realistic assessment under heterogeneous imaging conditions. The main novelty of this work is the integration of low-rank adaptation and adversarial domain alignment, demonstrating that robust cross-center generalization can be achieved without full-network fine-tuning, thereby enabling more scalable and resource-efficient deployment of Vision Transformer models in clinical practice.

This work introduces LoRA-GRL, a framework that integrates parameter-efficient low-rank adaptation with gradient-reversal-based domain harmonization for multi-center lung nodule classification from chest CT. The core contribution is a methodological demonstration that robust cross-center generalization can be achieved without full-network fine-tuning, suggesting a more scalable and computationally efficient pathway for evaluating Vision Transformers across heterogeneous imaging datasets. We validate our approach using patient-level five-fold cross-validation across three clinical centers on both Normal-versus-Benign and Normal-versus-Malignant tasks, and we systematically dissect the contributions of the GRL, adapter regularization, and CLAHE through comprehensive ablation analysis. While these internal multi-center results are encouraging and highlight the potential of the proposed strategy, we acknowledge that they constitute preliminary proof-of-concept evidence. We therefore refrain from claims of immediate clinical readiness and explicitly recognize that prospective external validation and real-world evaluation are necessary before any translational applications can be responsibly considered.

The remainder of this paper is organized as follows. Section 2 describes the materials and methods, including the study cohort, reference standard and labeling procedure, task formulation, preprocessing pipeline, model architecture, training objective, training configuration, and evaluation protocol. Section 3 presents the experimental results, including the main classification performance, per-center analysis, ablation study, and computational cost analysis. Section 4 discusses the findings and limitations of the study. Section 5 concludes the paper.

## 2. Materials and Methods

**Figure 1:**
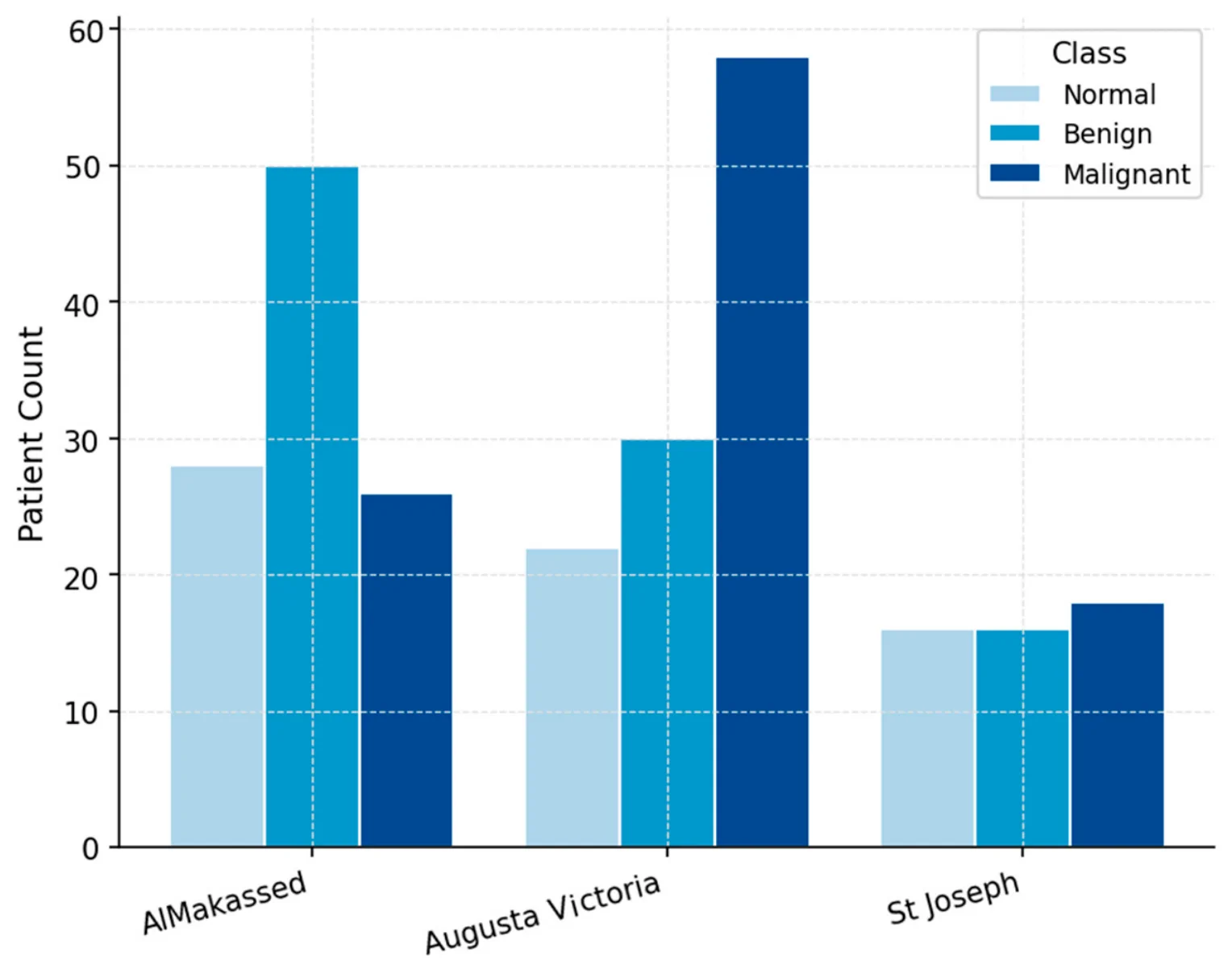
Dataset composition stratified by clinical center and diagnostic class.

The study cohort comprised 264 patients drawn from three clinical centers in Palestine: Al-Makassed Hospital (*n* = 104), Augusta Victoria Hospital (*n* = 110), and St. Joseph Hospital (*n* = 50). Patients were classified into three diagnostic categories: normal lung (*n* = 66; 25.0%), benign pulmonary nodule (*n* = 96; 36.4%), and malignant nodule (*n* = 102; 38.6%). Figure 1 presents the per-center class distribution.

For each center, we additionally report the number of CT volumes (patient studies) and the total number of axial slices, computed directly from the processed dataset (Table 1). The number of slices per volume is highly heterogeneous across centers, ranging from curated key-slice exports at one center to near-complete axial stacks at another, adding a further, previously unquantified dimension of the cross-center heterogeneity that the domain-adversarial (GRL) component is designed to address, alongside the scanner and protocol differences already reported in Table 1.

A formal prospective power analysis was not conducted; the sample size was determined by the available clinical data.

The three centers exhibited substantially different class profiles. Al-Makassed was benign-dominant (50 benign, 26 malignant, 28 normal), Augusta Victoria was malignant-dominant (58 malignant, 30 benign, 22 normal), and St. Joseph was approximately balanced (18 malignant, 16 benign, 16 normal). These distributional asymmetries, combined with inter-scanner technical variability, constitute the primary sources of domain shift that the Gradient Reversal Layer (GRL) component was designed to mitigate.

### 2.1. CT Scanner and Reconstruction Protocols

To characterize the imaging heterogeneity of the multicenter dataset, the CT scanner and acquisition/reconstruction parameters for each participating center are summarized in Table 1. Briefly, Al-Makassed Hospital used a Philips Incisive 128-slice multidetector CT scanner (Philips Healthcare, Amsterdam, The Netherlands) with iDose iterative reconstruction; St. Joseph Hospital used a Philips Incisive multidetector CT scanner (Philips Healthcare, Amsterdam, The Netherlands) with iDose iterative reconstruction; and Augusta Victoria Hospital used a Siemens Healthineers SOMATOM go.Top scanner (Siemens Healthineers, Erlangen, Germany) with SAFIRE iterative reconstruction. Tube voltage, detector collimation, pitch, gantry rotation time, and tube-current/dose modulation varied among centers and protocols, as did reconstruction slice thickness/increment, kernel/filter, matrix size, and display window settings.

### 2.2. Reference Standard and Labeling Procedure

All diagnostic labels were established through a structured dual-reader consensus protocol designed to minimize individual bias and enhance the reliability of the reference standard. Two board-certified thoracic radiologists independently reviewed each case, having full access to the patients’ imaging histories and prior reports. In the first stage, each radiologist examined the complete CT examination and identified the axial slice ranges that best demonstrated any suspicious pulmonary nodule. For every detected lesion, a provisional Lung-RADS v2022 category was assigned using the standardized ACR Lung-RADS calculator: category 1 (no nodules or definitively benign findings), category 2 (nodules with a very low probability of malignancy), category 3 (intermediate-risk nodules), category 4A (suspicious nodules), and category 4B (nodules highly suggestive of malignancy). Histopathological confirmation, where available, was also reviewed and recorded.

The present study frames the clinical tasks as two independent binary decisions (Normal vs. Benign and Normal vs. Malignant); therefore, the Lung-RADS assessments were subsequently mapped to three diagnostic classes using the following rule: cases assigned to Lung-RADS 1 were labelled Normal; cases assigned to Lung-RADS 2 were labelled Benign (provided no malignant features were suspected and no histological proof of malignancy existed); and cases assigned to Lung-RADS 4A or 4B, or those with biopsy-confirmed malignancy irrespective of the original Lung-RADS score, were labelled Malignant.

After the independent reads, the two radiologists convened for a consensus session during which any discrepant classifications were discussed and resolved. The final, agreed-upon label (Normal, Benign, or Malignant) for each patient was entered into the ground-truth database. This consensus-based, Lung-RADS-anchored labelling strategy ensures that every ground-truth annotation reflects a balanced clinical judgement, rather than the opinion of a single observer.

### 2.3. Task Formulation

Lung nodule classification is commonly formulated as a three-class problem involving Normal, Benign, and Malignant categories. Although this approach enables simultaneous prediction across all diagnostic classes, it does not fully reflect the clinical workflow followed during thoracic CT interpretation. In routine radiological practice, diagnostic assessment is hierarchical. Radiologists first determine whether an abnormal finding is present and subsequently evaluate its likelihood of malignancy. Consequently, nodule detection and malignancy assessment represent distinct diagnostic decisions rather than a single multi-class classification task.

To better align model development with this clinical decision-making process, the classification problem was decomposed into two independent binary tasks. The first task distinguished Normal from Benign cases (*N* = 162), representing the identification of clinically relevant pulmonary abnormalities. The second task distinguished Normal from Malignant cases (*N* = 168), focusing on the detection of malignant lesions. Each task was trained, validated, and evaluated independently using task-specific class balancing, augmentation strategies, and decision thresholds.

This formulation provides several methodological advantages. First, it aligns the learning objective with the sequential diagnostic pathway used in clinical practice. Second, it reduces class heterogeneity and minimizes ambiguity associated with simultaneous discrimination among biologically and clinically distinct categories. Third, it allows each classifier to focus on a specific diagnostic objective, promoting more targeted feature learning and potentially improving model discrimination. A direct Benign-versus-Malignant classification task was not included because it falls outside the primary screening pathway investigated in this study. Nevertheless, differentiating benign from malignant nodules remains clinically important and should be explored in future work. The proposed task decomposition therefore provides a clinically grounded framework for evaluating lung nodule classification in heterogeneous multi-center environments.

### 2.4. Preprocessing Pipeline

All DICOM CT volumes underwent an identical four-stage preprocessing pipeline regardless of diagnostic label, thereby preventing the introduction of any class-discriminative preprocessing artifact.

All CT volumes underwent a standardized preprocessing pipeline designed to preserve diagnostically relevant anatomical information while minimizing inter-center variability. First, a representative axial slice was selected by identifying the slice with the highest number of soft-tissue voxels within the Hounsfield Unit (HU) range of −200 to +200. This strategy preferentially samples the central thoracic region without requiring lesion-level annotations. Slice scoring was restricted to the central 60% of the field of view (20–80% of the axial extent in both dimensions) to avoid selecting artifact-prone slices near the pleural periphery, and a Gaussian weighting centered on the mid-volume position was applied to the voxel-count score to further discourage selection of extreme superior or inferior slices, where partial-volume and motion artifacts are more prevalent. To incorporate volumetric context without the computational burden of three-dimensional processing, axial, sagittal, and coronal slices centered on the selected location were extracted and resized to 224 × 224 pixels using bilinear interpolation. The three orthogonal views were then combined into a three-channel tensor, providing complementary anatomical information across multiple planes while maintaining compatibility with two-dimensional Vision Transformer architectures.

Prior to contrast enhancement, voxel intensities were clipped to the range [−1000, 400] HU, encompassing air, lung parenchyma, soft tissue, and bone, and linearly rescaled to [0, 1]. Subsequently, Contrast-Limited Adaptive Histogram Equalization (CLAHE) was applied independently to each channel using a clip limit of 1.0 and a tile size of 8 × 8. Recent lung-nodule CT classification work has likewise shown that CLAHE can improve local structural-detail visibility while limiting excessive contrast amplification [27]. This step enhanced local contrast and improved the visibility of nodule boundaries and textural characteristics while limiting excessive noise amplification. Although CLAHE was applied globally, its effect was primarily restricted to soft-tissue regions because CT windowing inherently down-weights voxel intensities outside the selected HU range, leaving background air and bone structures largely unaffected. Importantly, identical preprocessing was applied across all classes and clinical centers to avoid introducing class-specific or center-specific artifacts. Finally, pixel intensities were normalized using the ImageNet-21k channel means ([0.485, 0.456, 0.406]) and standard deviations ([0.229, 0.224, 0.225]), ensuring consistency with the input distribution expected by the pre-trained backbone network. The resulting tensors were stored as float32 arrays to reduce computational overhead during training.

To improve model generalization, online data augmentation was applied exclusively to the training set. Augmentation operations included random horizontal and vertical flips (*p* = 0.5), rotations within ±20°, affine scaling between 0.80 and 1.20, and color jitter transformations. No augmentation was applied to the validation or test sets beyond the fixed preprocessing pipeline, ensuring an unbiased assessment of model performance.

### 2.5. Model Architecture

**Figure 2:**
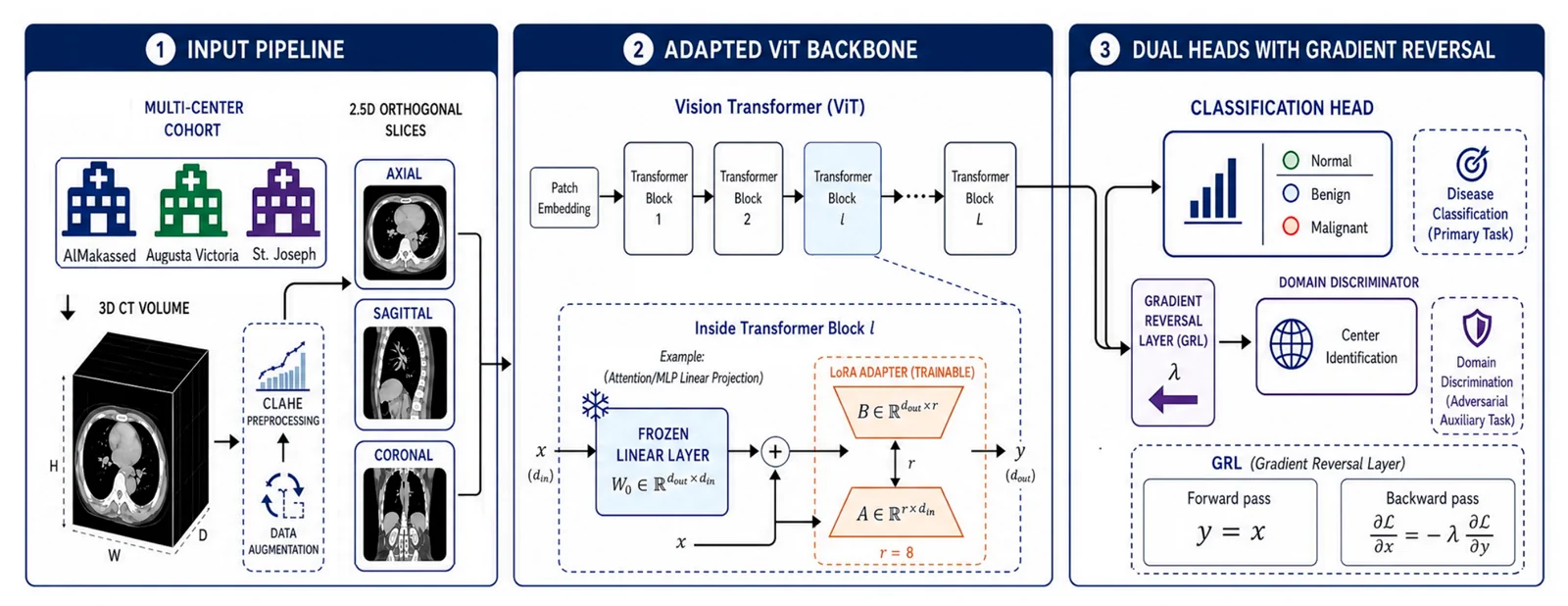
Overview of the LoRA-GRL framework: Multi-center CT volumes undergo CLAHE-based preprocessing and augmentation before axial, sagittal, and coronal views are combined as a 2.5D input. A frozen Vision Transformer with trainable rank-8 LoRA adapters produces a shared representation for diagnostic classification and GRL-based center discrimination.

Figure 2 illustrates the proposed LoRA-GRL framework, which comprises three components: a preprocessing pipeline, a frozen Vision Transformer backbone augmented with LoRA adapters, and two task-specific heads linked through a Gradient Reversal Layer (GRL). The following subsections describe each component in the order that data flows through the architecture.

The input pipeline begins with chest CT volumes acquired from three clinical centers (Al-Makassed, Augusta Victoria, and St. Joseph Hospital). Each volume undergoes CLAHE-based contrast enhancement and on-the-fly data augmentation. Three orthogonal slices (axial, sagittal, and coronal) are then extracted and stacked to form a 2.5-D input X ∈ ℝ^H×W×3^, which serves as the entry point to the feature extractor.

This input is processed by a ViT-S backbone (patch size 16, embedding dimension 384, 12 transformer blocks, 8 attention heads) pretrained on ImageNet-21k with AugReg augmentation and fine-tuned on ImageNet-1k [28]. All 21.1 M backbone parameters are frozen to preserve the visual representations learned during large-scale pretraining. The image is divided into non-overlapping patches, which are linearly projected into a token sequence. A learnable [CLS] token is prepended, positional embeddings are added, and the transformer blocks process the resulting sequence to produce a 384-dimensional [CLS] feature vector **f** ∈ ℝ^384^. The individual vector components are learned latent image features rather than predefined radiomic measurements and do not map one-to-one to a single physical descriptor; collectively, they encode combinations of intensity, contrast, texture, morphology, and spatial-context information across the three orthogonal CT views. Rather than updating the full backbone to adapt this representation to the target task, trainable LoRA modules are inserted in parallel with the linear projection layers throughout the transformer blocks. Each module decomposes the weight update into a product of two small matrices, learning task-specific corrections while keeping the number of trainable parameters to a fraction of the full model.

The resulting [CLS] feature vector is then shared by two downstream branches. The primary branch is a classification head that predicts the diagnostic category (normal, benign, or malignant). The auxiliary branch is a domain discriminator that predicts the originating clinical center. To encourage the backbone to produce center-invariant features, the domain discriminator is connected through a GRL, which acts as an identity during the forward pass but multiplies the gradient by −λ during backpropagation. This reversal forces the backbone to maximize domain confusion while the classification head minimizes diagnostic loss, eventually yielding a shared representation that is discriminative for nodule classification yet robust to inter-center differences in scanners, protocols, and acquisition settings.

Furthermore, the low-rank adapter matrices are injected in parallel with every linear projection layer in all transformer blocks, comprising the combined query-key-value (QKV) projection and the attention output (O) projection, as well as both linear layers of the feed-forward (MLP) sub-block, resulting in 48 adapter injection sites: the fused query–key–value (QKV) projection, the attention output projection, and both feed-forward layers (fc1 and fc2) of the MLP sub-block, i.e., four per block across all 12 transformer blocks. No adapters are placed on the patch or positional embeddings, the class token, the normalization layers, or the classification and domain heads. Extending adaptation to the MLP sub-blocks, rather than restricting it to the attention projections alone, was adopted because the MLP layers hold the majority of the backbone’s representational capacity, giving the adversarial domain-alignment objective sufficient reach to suppress center-specific signal beyond what attention-only adaptation permits, while the total LoRA parameter budget (0.590 M, 2.7% of the 21.1 M frozen backbone) remains small. For a target weight matrix $W_{0}\in R^{d_{out}\times d_{in}}$ (where $d_{in}$ is the input dimension and $d_{out}$ is the output dimension of the linear layer), the effective weight matrix $W_{eff}$ is defined as:

(1) $$W_{eff}=W+\frac{\alpha }{r}BA$$

where $A\in R^{r\times d_{in}}$ follows Kaiming-uniform initialization, and $B\in R^{d_{out}\times r}$ is initialized to zero. The rank r is set to 8, and the scaling factor α is 16. The zero initialization of B ensures that the adapter output is identically zero at the start of training, preserving the pre-trained backbone’s behavior during the critical early-learning phase. Only the parameters of A and B participate in gradient updates. The total LoRA parameter count is 0.590 M across all injection sites [22]. Together with the classification head (0.099 M) and the domain head (0.099 M), this yields 0.788 M (reported throughout as 0.789 M after rounding) total trainable parameters.

The classification head, denoted as $C\left(f;\theta_{c}\right)$, is a two-layer MLP that maps the 384-dimensional CLS token feature vector f to binary logits. It consists of Linear (384, 256), ReLU activation, Dropout (*p* = 0.3), and Linear (256, 2). The output logits are then passed through a sigmoid function to produce class probabilities $p_{cls}\in \left[0,1\right]$. This head contributes 0.099 M trainable parameters, collectively represented by $\theta_{c}$, and follows Kaiming-uniform initialization.

The domain classification heads, denoted as $D\left(f;\theta_{d}\right)$, maps the 384-dimensional CLS token feature vector f to logits representing the probability of origin from one of three institutions. It consists of Linear (384, 256), LayerNorm (256), ReLU, Dropout (*p* = 0.2), and Linear (256, 3). This head is connected to the backbone through a Gradient Reversal Layer (GRL) [16]. In the forward pass, the GRL acts as an identity function:

(2) $$f_{GRL}\left(x\right)=x$$

In the backward pass, the GRL multiplies the incoming gradient by negative λ before transmitting it to the backbone:

(3) $$\frac{\partial L}{\partial x}=-\lambda \frac{\partial L}{\partial y}$$

where $\lambda \in \left[0,1\right]$ increases linearly from 0 to 1 over the first 50% of training iterations. This progressive warmup schedule prevents the adversarial signal from destabilizing classification learning before the classification head has sufficiently converged. The inclusion of Layer Normalization in the domain head mitigates gradient magnitude instability when mini-batches contain skewed center compositions. The domain head contributes 0.099 M trainable parameters. At inference time, the GRL is an identity function, ensuring that LoRA-GRL imposes no additional computational overhead or latency compared to plain LoRA.

### 2.6. Training Objective

The total training objective combines three distinct loss components, each targeting a specific aspect of the learning process:

(4) $$L=L_{cls}+\lambda_{d}L_{dom}+\lambda_{r}\sum_{l,m}∥B_{lm}A_{lm}{∥}_{F}^{2}$$

Here, $L_{cls}$ represents the binary cross-entropy (BCE) classification loss [29], serving as the primary supervised signal for the nodule classification task. For a given input x, true label $y_{true}\in \{0,1\}$, and predicted probability $p_{cls}=\sigma \left(C\left(f\left(x\right);\theta_{c}\right)\right)$, the BCE loss is defined as:

(5) $$L_{cls}=-\frac{1}{N}\sum_{i=1}^{N}\left[y_{true,i}log\left(p_{cls,i}\right)+\left(1-y_{true,i}\right)log\left(1-p_{cls,i}\right)\right]$$

$L_{dom}$ is the cross-entropy (CE) domain classification loss, computed on GRL-transformed features [16,30,31]. For a given input x, the true domain label $d_{true}\in \{1,2,3\}$, and predicted domain probabilities $p_{dom}=\mathrm{softmax}\left(D\left(f_{GRL}\left(x\right);\theta_{d}\right)\right)$, the CE loss is defined as:

(6) $$L_{dom}=-\frac{1}{N}\sum_{i=1}^{N}\sum_{j=1}^{3}y_{dom,ij}log\left(p_{dom,ij}\right)$$

Because the GRL negates its gradient during backpropagation, minimizing $L_{dom}$ from the perspective of the backbone effectively maximizes the domain discriminator’s confusion, thereby driving the backbone towards learning center-invariant representations. The third term, $\sum_{l,m}∥A_{lm}B_{lm}{∥}_{F}^{2}$, is a Frobenius-norm regularization applied across all l=48 adapter injection sites (indexed by l,m). This term applies a rank-aware penalty to large adapter corrections, preventing overspecialization to the training center distribution. The hyperparameters $\lambda_{d}=0.5$ and $\lambda_{r}=5\times 10^{-5}$ were selected by grid search on a held-out development fold.

### 2.7. Training Configuration

Optimization is performed using AdamW with a learning rate of $3\times 10^{-4}$ applied to all trainable parameters (adapters, classification head, domain head) [20,30], a weight decay of $1\times 10^{-2}$, and a cosine annealing schedule with a maximum of 100 epochs, over which the learning rate decayed to a minimum of 1% of its initial value. The batch size is set to 8. Within each training fold, the minority class is oversampled to match the majority class count. This oversampling is performed using independent on-the-fly augmentation per sample, ensuring that the model never encounters an identical (image, augmentation) pair twice. Early stopping is employed based on an Exponential Moving Average (EMA)-smoothed validation AUC, with an EMA coefficient of 0.3, a patience of 20 epochs, and a minimum of 30 training epochs. All experiments are conducted with a fixed random seed (seed 42) and the cuBLAS workspace configured for reproducibility. The complete training procedure is summarized in Algorithm 1.
**Algorithm 1:** LoRA-GRL Training ProcedureInput:    CT volumes {*V_i_*} with labels *y_i_* (class) and *d_i_* (center),               *i* = 1, …, *N* patients from *C* = 3 clinical centers Output: Trained classification head *C*(·; *θ*_c_) and LoRA-adapted backbone STAGE 1: Preprocessing (per patient i) 1:    s* ← argmax_s_ Σ 1[−200 ≤ HU(*x*,*y*,*s*) ≤ 200]·*G*(*s*; *μ* = center, *σ*)               // best axial slice: soft-tissue voxel count, central−60% ROI,               // Gaussian-weighted toward mid-volume
2:    *X_ax_*, *X_sag_*, *X_cor_* ← extract orthogonal slices through s*
3:    for each view *v*
∈ {ax, sag, cor}:
4:           *X_v_* ← clip(*X_v_*, −1000, 400)                           // HU windowing
5:           *X_v_* ← rescale(*X_v_*, [0, 1])
6:           *X_v_* ← resize(X_v_, 224 × 224, bilinear)
7:           *X_v_* ← CLAHE(*X_v_*, clip_limit = 1.0, tile = 8×8)
8:    *X_i_* ← stack(*X_ax_*, *X_sag_*, *X_cor_*)                         // 2.5-D, 224 × 224 × 3
9:    *X_i_* ← normalize(*X_i_*, *μ*_ImageNet_, *σ*_ImageNet_)
10:  (train only) *X_i_* ← augment(*X_i_*) STAGE 2: Model Construction
11:  *f_θ_* ← ViT-S/16, pretrained on ImageNet-21k + 1k, all 21.1 M params FROZEN
12:  for each Linear layer l ∈ {qkv, proj, fc1, fc2} across all 12 blocks:               // 48 injection sites total 13:          *A_l_* ~ KaimingUniform(shape = *r* × *d*_in_),    *r* = 8
14:          *B_l_* ← 0, shape = *d*_out_ × *r*
15:          *W*_*eff*,*l*_ ← *W_l_* + (*α*/*r*)·*B_l_ A_l_*, *α* = 16
16:  *C*(·; *θ*_c_) ← Linear(384, 256) → ReLU → Dropout(0.3) → Linear(256, *n*_classes_)
17:  *D*(·; *θ*_d_) ← Linear(384, 256) → LayerNorm(256) → ReLU → Dropout(0.2)                                → Linear(256, *n*_centers_)
18:  GRL(*x*) ← *x*                                                            // forward: identity        *∂*GRL/*∂x* ← −*λ*·*∂L/∂x*                                           // backward: gradient reversal STAGE 3: Training (5-fold StratifiedGroupKFold, patient-level) 19:  for fold = 1 to 5: 20:          split patients into outer-train/outer-test, grouped by patient ID               split outer-train patients into inner-train (80%) and validation (20%)               // outer-test remains untouched during training and early stopping 21:          for epoch = 1 to 100: 22:                 *λ* ← min(1, 2·epoch/100)                        // linear warm-up, reaches 1 at 50% of training 23:                 for each mini-batch B (size = 8): 24:                        *f* ← *f*_*θ*_(*X*)                               // shared 384-dim CLS feature 25:                        *p*_cls_ ← *σ*(*C*(*f*; *θ*_c_)) 26:                        *p*_dom_ ← softmax(D(GRL*_λ_*(*f*); *θ*_d_)) 27:                        *L*_cls_ ← BCE(*p*_cls_, *y*) 28:                        *L*_dom_ ← CE(*p*_dom_, *d*) 29:                        *L*_reg_ ← Σ*_l_*_=1_^48^ ‖*B_l_ A_l_*‖^2^_F_ 30:                        *L* ← *L*_cls_ + *λ*_d_·*L*_dom_ + *λ*_r_·*L*_reg_                               (*λ*_d_ = 0.5, *λ*_r_ = 5 × 10^−5^) 31:                        update {*A_l_*, *B_l_*}*_l_*_=1_^48^, *θ*_c_, *θ*_d_    via AdamW                                (lr = 3 × 10^−4^, wd = 1 × 10^−2^, cosine anneal to 1% of lr) 32:                 compute EMA-smoothed validation AUC (*β* = 0.3) 33:                 if epoch ≥ 30 and no improvement for 20 epochs: break 34:          evaluate on held-out test fold → record AUC, Sens., Spec., F1, AUC Return: {*A_l_*, *B_l_*}, *θ*_c_      (*θ*_d_ and GRL discarded—inference-time no-op)

### 2.8. Evaluation Protocol

Model performance was evaluated using 5-fold StratifiedGroupKFold cross-validation with patient-level grouping constraints. In each fold, approximately 80% of patients were used for model training and 20% were held out for testing. The exact per-fold allocation varied slightly to preserve both patient-level grouping and class stratification. This strategy ensured that all DICOM slices belonging to a given patient were assigned exclusively to either the training or testing partition within each fold, thereby preventing data leakage arising from the strong spatial correlation between adjacent CT slices. The primary performance metric was the pooled Area Under the Receiver Operating Characteristic Curve (AUC), calculated from the concatenated test predictions across all folds.

To assess the consistency of model performance, the mean and standard deviation of each metric were also reported across folds. Secondary evaluation metrics included Weighted F1-Score (WF1), Accuracy (ACC), Sensitivity (recall of the disease class), and Specificity (recall of the normal class). In addition, per-center AUC values were computed using pooled test predictions stratified by the originating clinical institution to evaluate the effectiveness of the proposed framework under heterogeneous multi-center conditions.

To complement these metrics and to account for class imbalance, we additionally report Precision, the Matthews correlation coefficient (MCC), and the area under the precision–recall curve (PR-AUC). For a given positive class, Precision = TP/(TP + FP); MCC = (TP·TN − FP·FN)/√[(TP + FP)(TP + FN)(TN + FP)(TN + FN)], a single balanced summary of the confusion matrix on a [−1, 1] scale that is robust to class skew; and PR-AUC is the area under the precision–recall curve. All three are computed from the same pooled cross-validation predictions used for the other metrics, so no additional training is required. Results for the normal-versus-malignant and normal-versus-benign tasks are given in Table 2 and Table 3, respectively.

Statistical comparisons between methods were performed on the pooled cross-validation predictions, the held-out predictions of all five folds concatenated, with the individual patient as the unit of analysis. For a given pair of methods, the test statistic was the absolute difference in pooled macro ROC-AUC. The null distribution was constructed by a paired permutation test: over 1000 permutations (fixed random seed), each patient’s pair of prediction vectors was independently swapped between the two methods with probability 0.5, the two AUCs were recomputed, and their absolute difference was recorded; the two-sided empirical *p*-value is the proportion of permutation differences whose magnitude is at least the observed |ΔAUC|. Comparisons were pre-specified for LoRA-GRL against plain LoRA and against Full-FT on each of the two binary tasks, four comparisons in total, and raw *p*-values were Bonferroni-corrected by a factor of four (significance threshold 0.05/4 = 0.0125). As the effect size, we report the AUC difference (ΔAUC) for each comparison, together with 95% confidence intervals obtained by patient-level percentile bootstrap (10,000 resamples) on both the pooled AUCs and their paired differences. Given the modest multi-center sample size, these tests are interpreted as supportive rather than definitive inferential evidence.

### 2.9. Interpretability Analysis

To provide a qualitative, exploratory assessment of the image regions driving the model’s predictions, we applied Grad-CAM adapted to the Vision Transformer backbone. Because the classification head pools only the class (CLS) token, the final transformer block’s patch-token outputs receive no gradient from the CLS-pooled objective and are unsuitable as a Grad-CAM target; we therefore compute Grad-CAM at the output of the second-to-last transformer block, whose patch tokens receive nonzero gradient through the final block’s self-attention. Channel weights were obtained as the spatial mean of the gradient of the target-class score with respect to the block’s patch-token activations; the class-activation map was formed as the ReLU of the weighted sum of these activations, reshaped from the 196 patch tokens to a 14 × 14 grid and upsampled to 224 × 224 for overlay on the input axial slice. Grad-CAM is used here as a qualitative saliency tool rather than a validated localization metric.

### 2.10. Computational Cost and Inference Latency Measurement

Inference latency was measured as the wall-clock time of a single forward pass at batch size 1 (the per-scan deployment case), averaged over 300 timed iterations after 30 warm-up iterations. For LoRA and LoRA-GRL, latency was measured both with the adapters kept as a parallel branch (unmerged) and with the adapters folded into the frozen backbone weights, W′ = W + (α/r)·A·B (the standard merged-deployment configuration); the merged configuration is reported as the deployed inference time.

## 3. Experimental Results

### 3.1. Main Classification Performance

Table 4 summarizes the classification performance. As shown, for the Normal vs. Benign task, LoRA-GRL achieved an AUC of 0.9735 (only 0.0012 lower than Full-FT) while attaining the highest specificity (0.9667) and reducing trainable parameters by 96.4%. For Normal vs. Malignant, LoRA-GRL obtained the numerically highest AUC (0.9869), weighted F1-score (0.9286), accuracy (0.9286), and sensitivity (0.9412), with a 5.9 percentage point sensitivity gain over Full-FT and unchanged specificity (0.9697). Both LoRA variants used only 0.789 M trainable parameters (3.6% of Full-FT). Adding adversarial domain harmonization (GRL) further improved malignant nodule discrimination compared with standard LoRA. Training time per epoch was 1.1–1.2 s (vs. 0.8 s for Full-FT), with no inference overhead.

**Figure 3:**
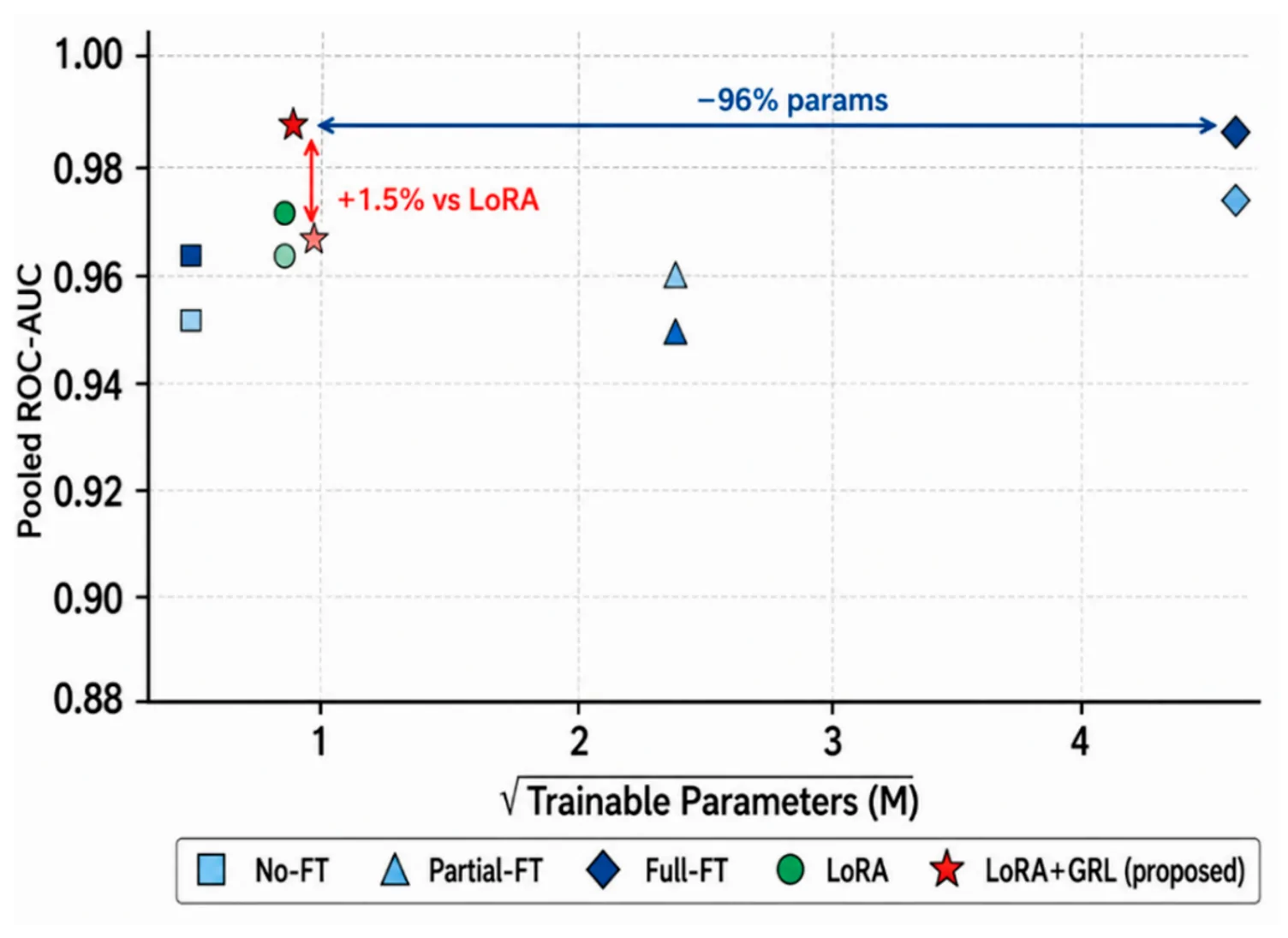
Performance-efficiency trade-off: pooled AUC plotted against the square root of the number of trainable parameters for both diagnostic tasks. Light-colored points represent the Normal-versus-Benign task and dark-colored points represent the Normal-versus-Malignant task; points closer to the upper-left indicate higher pooled AUC with fewer trainable parameters.

The parameter-efficiency trade-off is further illustrated in Figure 3: LoRA-GRL achieved the highest malignant AUC and a benign AUC within 0.0012 of Full-FT, using only 0.789 M parameters, placing it in the most favorable region of the performance-efficiency space.

The distribution of the data points indicates that LoRA-GRL occupies the most favorable region of the performance efficiency space, combining high predictive accuracy with a substantial reduction in model complexity. These findings indicate that integrating adversarial domain harmonization into a parameter-efficient adaptation framework improves classification performance without increasing the number of trainable parameters, thereby providing a more computationally efficient solution for multi-center lung nodule classification.

**Figure 4:**
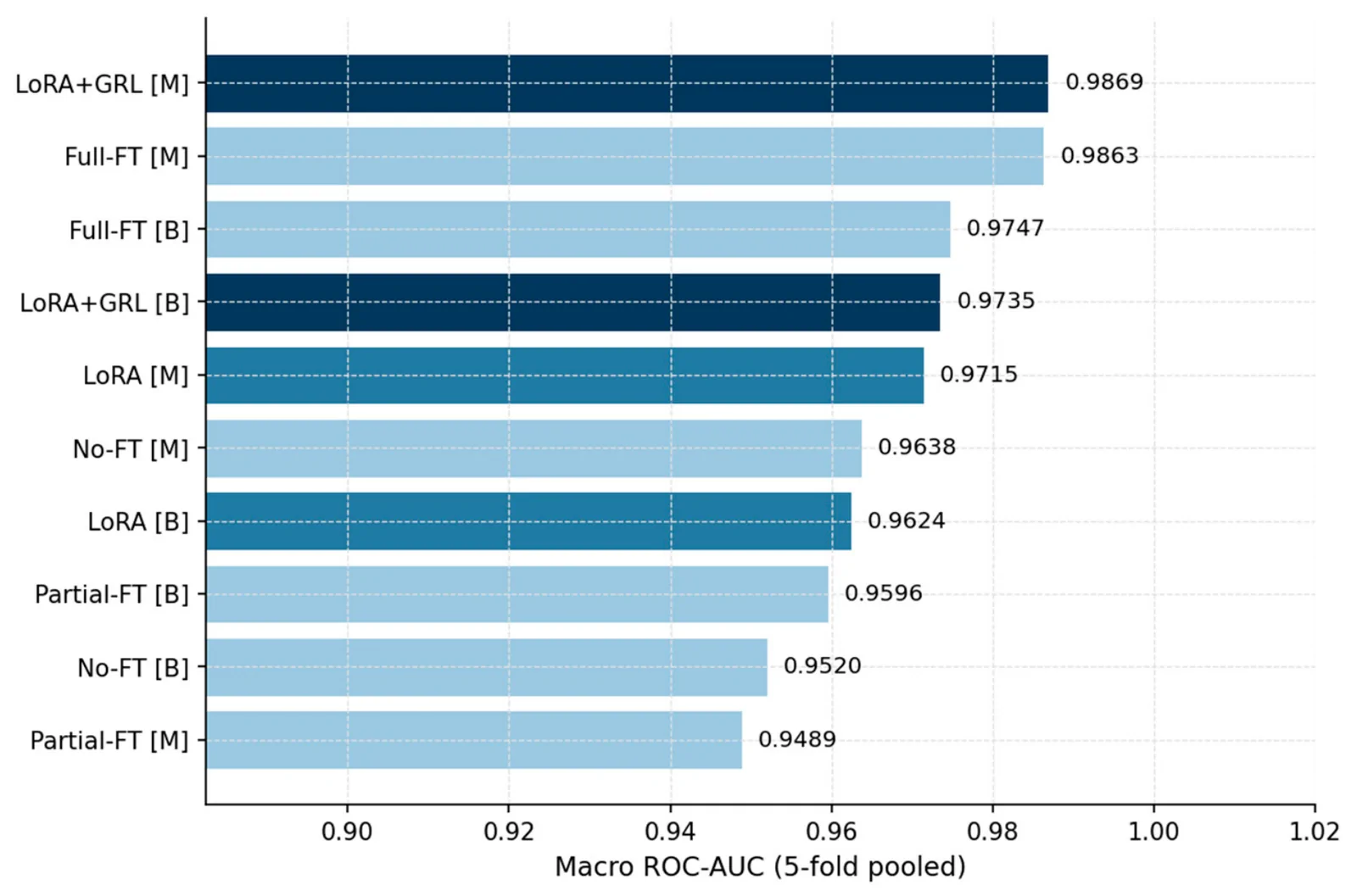
Ranked pooled macro ROC-AUC for each method-task combination across five folds. [B] denotes the Normal-versus-Benign task and [M] the Normal-versus-Malignant task; darker bars highlight LoRA-GRL.

As depicted in Figure 4 and consistent with Table 4, LoRA-GRL ranked highest or second-highest in AUC for both tasks and was numerically higher than conventional LoRA by approximately 1.5 percentage points in malignant classification without additional parameters; as detailed below, however, this and the other pairwise differences fell within overlapping confidence intervals and did not reach statistical significance.

The comparison also highlights the benefit of integrating adversarial domain harmonization with LoRA. While conventional LoRA achieved AUCs of 0.9624 and 0.9715 for the Benign and Malignant tasks, respectively, the proposed LoRA-GRL framework improved these values to 0.9735 and 0.9869 without increasing the number of trainable parameters. The improvement was particularly evident for malignant nodule classification, where the AUC increased by approximately 1.5 percentage points, a difference that did not reach statistical significance (Bonferroni-adjusted *p* = 0.08).

Across the five cross-validation folds, the LoRA-GRL-versus-LoRA improvements were directionally consistent; for the Normal-versus-Malignant task, the pooled AUC gain was +0.0154 and the paired fold-level permutation analysis yielded a Bonferroni-adjusted *p* = 0.08. Given the modest cohort and the limited number of correlated folds, this did not meet the conventional threshold for statistical significance and is interpreted as a potentially clinically relevant, hypothesis-generating effect requiring confirmation in a larger external cohort.

In contrast, No-FT and Partial-FT consistently produced lower AUC values across both tasks, indicating that freezing the backbone without effective adaptation or updating only a subset of transformer layers was insufficient to fully capture the domain-specific characteristics of the multi-center dataset. Collectively, these findings show that LoRA-GRL achieves classification performance comparable to conventional full fine-tuning while retaining the computational efficiency of parameter-efficient adaptation.

**Figure 5:**
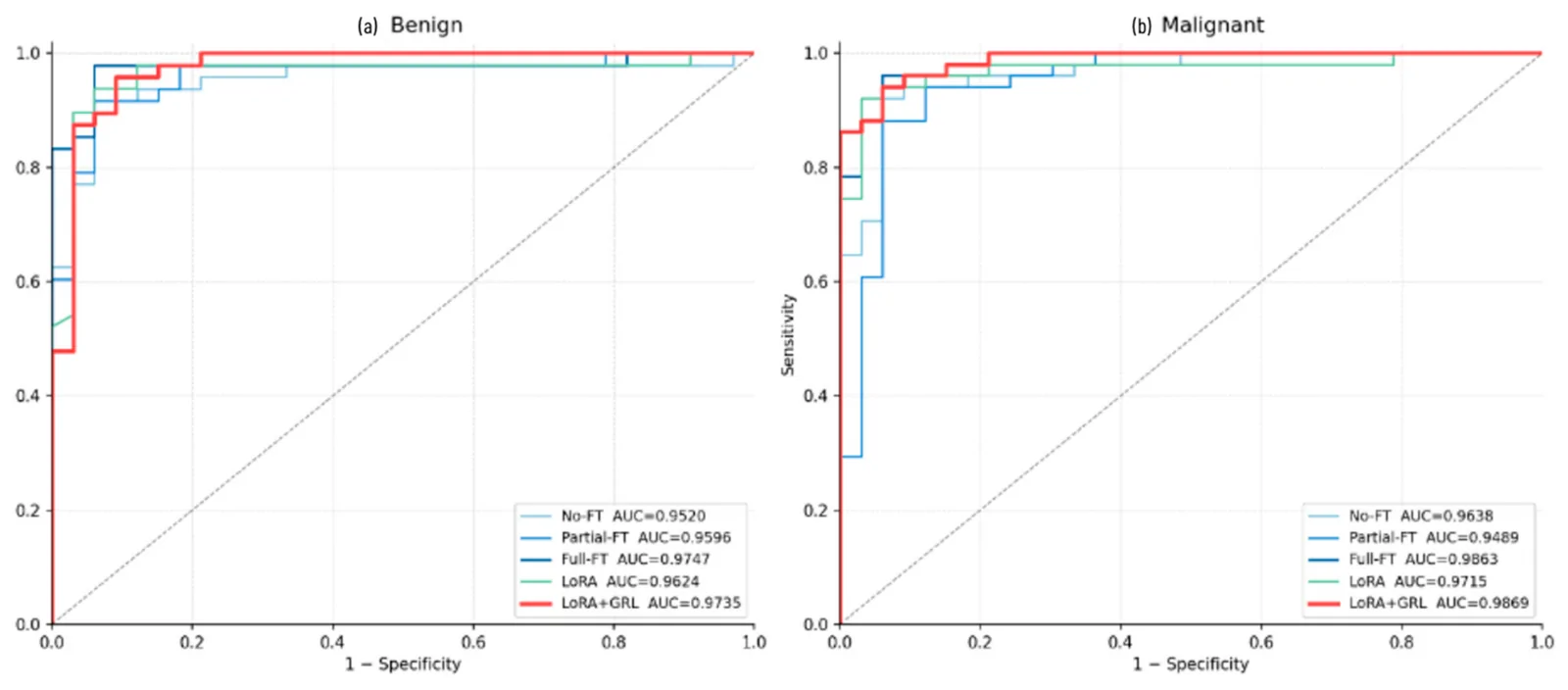
Receiver operating characteristic curves for all five methods on (**a**) Benign and (**b**) Malignant tasks, pooled across five test folds.

ROC analysis (Figure 5) confirmed that LoRA-GRL showed high observed discrimination, with a steep initial rise for malignant classification indicating high sensitivity at low false-positive rates, and benign-task AUC nearly identical to Full-FT.

For the Normal versus Benign task (Figure 5a), LoRA-GRL achieved an AUC of 0.9735, closely approaching the performance of Full-FT (0.9747) while clearly outperforming LoRA (0.9624), Partial-FT (0.9596), and No-FT (0.9520). The near overlap between the LoRA-GRL and Full-FT curves indicates that parameter-efficient adaptation preserved almost the same discriminative capability as full-network fine-tuning despite requiring substantially fewer trainable parameters.

A similar pattern was observed for the Normal versus Malignant task (Figure 5b). LoRA-GRL achieved an AUC of 0.9869, comparable to Full-FT (0.9863) and showing a more noticeable improvement over conventional LoRA (0.9715), Partial-FT (0.9489), and No-FT (0.9638). The steeper initial rise of the LoRA-GRL ROC curve indicates higher sensitivity at low false-positive rates, suggesting improved identification of malignant nodules without compromising specificity.

Pre-specified comparisons of LoRA-GRL versus plain LoRA and versus Full-FT were evaluated on both tasks using patient-level permutation tests and patient-level bootstrap 95% confidence intervals. After Bonferroni correction, none of the AUC differences were statistically distinguishable; the paired differences were small and all confidence intervals included zero.

Across both classification tasks, the ROC analysis demonstrates that incorporating adversarial domain harmonization into the LoRA framework consistently improved discrimination while maintaining the computational advantages of parameter-efficient fine-tuning. The largest performance gain was observed for malignant nodule classification, where the proposed framework achieved among the highest discriminative performance of all evaluated methods, within overlapping confidence intervals.

### 3.2. Per-Centre Analysis

Table 5 summarizes the classification performance across the three participating clinical centers. While all methods exhibited some degree of performance variability between institutions, LoRA-GRL consistently achieved the highest or near-highest AUC values across centers, indicating improved robustness to inter-center differences in image acquisition and patient distribution.

For the Normal versus Benign task, LoRA-GRL achieved the highest mean AUC (0.9807), outperforming Full-FT (0.9750), Partial-FT (0.9693), LoRA (0.9639), and No-FT (0.9552). Although Full-FT produced the highest AUC at AlMakassed Hospital (0.9857), LoRA-GRL achieved the best performance at Augusta Victoria Hospital (0.9879) and St. Joseph Hospital (0.9922), resulting in the highest overall cross-center average.

The advantage of LoRA-GRL was more noticeable for the Normal versus Malignant task. The proposed framework achieved the highest AUC at AlMakassed Hospital (0.9945) and St. Joseph Hospital (0.9941), while its performance at Augusta Victoria Hospital (0.9934) was nearly identical to that of conventional LoRA (0.9937). Consequently, LoRA-GRL achieved the highest mean AUC (0.9940), comparable to Full-FT (0.9861) and substantially outperforming Partial-FT (0.9692), LoRA (0.9649), and No-FT (0.9775).

In addition to achieving the highest average performance, LoRA-GRL exhibited the greatest consistency across institutions. For the Normal versus Malignant task, the difference between the highest and lowest center-specific AUC was only 0.0011, representing the smallest cross-center variation among all evaluated methods. In contrast, conventional LoRA showed a range of 0.0760, Partial-FT 0.0265, Full-FT 0.0091, and No-FT 0.0225. The substantially lower inter-center variability indicates that the proposed adversarial domain harmonization effectively reduced center-specific performance fluctuations while maintaining high classification accuracy across heterogeneous CT acquisition environments.

### 3.3. Ablation and Sensitivity Analysis

Table 6 presents the ablation analysis for the Normal versus Malignant classification task, evaluating the individual contributions of adversarial domain harmonization (GRL), LoRA regularization, and CLAHE-based contrast enhancement. All LoRA-based configurations maintained an identical parameter budget (0.789 M), ensuring that performance differences resulted solely from the inclusion of additional model components.

The baseline LoRA model achieved an AUC of 0.9715, serving as the reference parameter-efficient configuration. Incorporating the Gradient Reversal Layer increased the AUC to 0.9792, while also improving weighted F1-score (0.9203), accuracy (0.9226), and sensitivity (0.9020). These results indicate that adversarial domain harmonization enhanced feature generalization across heterogeneous imaging centers without increasing the number of trainable parameters.

Introducing Frobenius-norm regularization further improved performance, increasing the AUC to 0.9821 and sensitivity to 0.9216. This improvement indicates that regularizing the LoRA adapters reduced excessive adaptation to center-specific characteristics while preserving discriminative information relevant to malignant nodule detection. The final addition of CLAHE-based contrast enhancement yielded the best overall performance. The complete LoRA-GRL framework achieved an AUC of 0.9869, weighted F1-score of 0.9286, accuracy of 0.9286, and sensitivity of 0.9412, exceeding all intermediate configurations while maintaining the same parameter count.

Compared with the baseline LoRA model, the complete framework improved the AUC by 1.54 percentage points and sensitivity by 5.88 percentage points, demonstrating the complementary contributions of domain harmonization, adapter regularization, and image enhancement. Notably, LoRA-GRL achieved an AUC comparable to the Full-FT reference (AUC = 0.9869 versus 0.9863) despite optimizing only 0.789 million trainable parameters compared with 21.865 million. These findings indicate that the performance gains resulted from the synergistic integration of the proposed components rather than from increased model capacity.

We further examined the framework’s sensitivity to its two principal hyperparameters, the LoRA rank r and the GRL coefficient $\lambda_{d}$, using a local subset of the Normal-versus-Malignant task (Table 7). Holding all other settings fixed, we varied r∈{4, 8, 16} at $\lambda_{d}=0.5$ and $\lambda_{d}\in \{0.1,\;0.5,\;1.0\}$ at r=8, with each configuration evaluated using patient-level five-fold cross-validation.

Performance was relatively robust to changes in LoRA rank, with the intermediate rank r=8 providing the strongest overall balance between predictive performance and parameter efficiency. At $\lambda_{d}=0.5$, increasing the rank from r=4 to r=8 improved pooled AUC from 0.848 to 0.913, together with increases in weighted F1 score from 0.749 to 0.868, sensitivity from 0.760 to 0.860, specificity from 0.723 to 0.867, PR-AUC from 0.906 to 0.945, and MCC from 0.481 to 0.730. Increasing the rank further to r=16 did not yield additional gains, with AUC decreasing slightly to 0.897 and corresponding reductions in weighted F1 score, sensitivity, PR-AUC, and MCC. Given that the number of trainable adapter parameters increased from 0.74 M at r=8 to 1.33 M at r=16, these results indicate diminishing returns from increasing LoRA capacity beyond the intermediate rank.

In contrast, performance showed greater sensitivity to the strength of the GRL coefficient. At r=8, the lowest adversarial weighting, $\lambda_{d}=0.1$, achieved the highest pooled AUC of 0.947, together with the highest PR-AUC (0.961), weighted F1 score (0.891), sensitivity (0.920), and MCC (0.773). Increasing $\lambda_{d}$ to 0.5 reduced AUC moderately to 0.913 but produced a more balanced sensitivity and specificity profile, with values of 0.860 and 0.867, respectively, compared with 0.920 and 0.833 at $\lambda_{d}=0.1$. Stronger gradient reversal at $\lambda_{d}=1.0$ resulted in a further decline in performance, with AUC decreasing to 0.876, weighted F1 score to 0.797, and MCC to 0.588. The larger fold-to-fold AUC variability observed at $\lambda_{d}=1.0$ (SD = 0.071) also suggests reduced stability under stronger adversarial regularization.

The sensitivity analysis indicates that the framework is comparatively robust to moderate variation in LoRA rank but more responsive to the strength of domain-adversarial regularization. An intermediate LoRA rank of r=8 provided an efficient balance between adapter capacity and predictive performance, whereas relatively mild GRL regularization was sufficient to achieve strong discrimination. Although $\lambda_{d}=0.1$ yielded the highest overall AUC, the reference configuration of r=8 and $\lambda_{d}=0.5$ retained strong discrimination while providing the most balanced sensitivity–specificity profile and a modest adapter budget.

### 3.4. Comparison with Competing Methods

The comparison with competing methods showed that Full-FT achieved the highest overall predictive performance, with a pooled AUC of 0.953, weighted F1 of 0.916, and MCC of 0.825, but required optimization of 21.814 million parameters (Table 8). Among the parameter-efficient methods, Houlsby Adapters achieved the strongest discrimination (AUC = 0.942), while the proposed LoRA-GRL achieved an AUC of 0.921 using only 0.739 million trainable parameters.

The most direct comparison was with conventional DANN, which used the same gradient-reversal objective but required full-network fine-tuning. LoRA-GRL achieved comparable performance to DANN (AUC 0.921 vs. 0.917) while using approximately 29.5-fold fewer trainable parameters. It also achieved higher weighted F1 (0.857 vs. 0.844), specificity (0.867 vs. 0.833), PR-AUC (0.957 vs. 0.938), and MCC (0.708 vs. 0.680). This indicates that domain-adversarial learning can be incorporated effectively through low-rank adaptation without requiring full-backbone optimization.

Compared with plain LoRA, LoRA-GRL produced similar AUC (0.921 vs. 0.925) but higher specificity (0.867 vs. 0.800) and PR-AUC (0.957 vs. 0.938), although sensitivity was lower (0.840 vs. 0.920). The results indicate that the main advantage of LoRA-GRL is parameter-efficient domain adaptation rather than superior absolute classification performance. Because the confidence intervals overlapped across several methods, the observed differences should be interpreted as comparative trends rather than statistically significant superiority.

The paired bootstrap analysis showed that none of the observed AUC differences between LoRA-GRL and the competing methods reached statistical significance (Table 9). All 95% confidence intervals for the paired AUC differences included zero, with bootstrap *p*-values ranging from 0.182 to 0.874. Although Full-FT and Houlsby Adapters showed numerically higher AUCs than LoRA-GRL, the corresponding differences were small and not statistically distinguishable at the current sample size. Similarly, LoRA-GRL showed slightly higher AUCs than DANN, BitFit, and VPT, but these differences were also non-significant.

These findings indicate that the methods achieved broadly comparable discrimination on the development subset. Importantly, LoRA-GRL performed statistically comparably to full-network DANN and Full-FT despite using only 0.739 million trainable parameters, reinforcing its advantage as a parameter-efficient adaptation strategy rather than demonstrating statistically superior classification performance.

Table 10 places the proposed LoRA-GRL framework in the context of recent lung nodule classification studies. The present approach achieved an AUC range of 0.9735–0.9869, which is comparable to or higher than the values reported by recent transformer-based methods, including LoRA-based SwinV2-b (AUC = 0.970), full fine-tuning with DCSwinB (AUC = 0.94), and the hybrid C-Swin model (AUC = 0.93–0.97).

A key distinction of the proposed framework is that it combines parameter-efficient fine-tuning with explicit domain adaptation. Unlike the competing studies, which were evaluated primarily on single benchmark datasets and did not incorporate domain-adversarial learning, this study used data from three independent clinical centers with heterogeneous class distributions and scanner characteristics. Despite these additional sources of domain variability, LoRA-GRL maintained strong discrimination while requiring only 0.789 million trainable parameters.

These findings indicate that the proposed approach achieves competitive classification performance while simultaneously addressing parameter efficiency and inter-center domain shift. However, direct numerical comparisons should be interpreted cautiously because the studies used different datasets, patient populations, preprocessing pipelines, validation strategies, and classification tasks.

The slice-coverage analysis showed that the annotation-free selection heuristic captured the radiologists’ recorded nodule range in 164 of 198 matched nodule cases (82.8%) (Table 11). Coverage was 75.0% for benign nodules (72/96) and 90.2% for malignant nodules (92/102).

Twenty cases were identified in which the radiologist-annotated region was not present in the reconstruction or series supplied to the analysis pipeline. When the analysis was restricted to the 178 cases in which the annotated region was available in the pipeline input, overall coverage increased to 92.1% (164/178). Within this evaluable subset, the selected slice fell within the recorded nodule range in 88.9% of benign cases (72/81) and 94.8% of malignant cases (92/97).

These findings provide strong quantitative support for the annotation-free slice-selection strategy, demonstrating that it captured the clinically relevant nodule region in more than 90% of evaluable cases. The difference between the raw coverage of 82.8% and the evaluable-case coverage of 92.1% also indicates that a meaningful proportion of apparent failures resulted from reconstruction or series-selection inconsistencies rather than from the slice-selection heuristic itself. Coverage remained somewhat higher for malignant than benign nodules, although both groups showed high overlap when the relevant annotated region was available to the pipeline.

### 3.5. Computational Cost Analysis

Beyond the reduction in trainable parameters, the practical efficiency of LoRA-GRL can be quantified in terms of storage and memory footprint. At fp32 precision, a LoRA-GRL adapter checkpoint requires only 3.16 MB, compared to 87.46 MB for a fully fine-tuned model checkpoint, a 96.4% reduction consistent with the trainable parameter reduction. This has direct implications for multi-site clinical deployment, where a single shared frozen backbone (84.4 MB) can be combined with lightweight per-institution or per-task adapters, rather than maintaining a separate full model copy for each deployment context. For example, deploying five site- or task-specific model variants would require approximately 100.2 MB under the LoRA-GRL scheme (one shared backbone plus five adapters), compared to approximately 437.3 MB for five independently fully fine-tuned models. In addition, since the AdamW optimizer maintains two auxiliary state tensors per trainable parameter, the optimizer memory footprint for trainable parameters is reduced from 262.38 MB under full fine-tuning to 9.47 MB under LoRA-GRL, lowering GPU memory requirements during training. Training time per epoch (1.1–1.2 s) is reported in Table 4 and is not reduced relative to full fine-tuning (0.8 s), as the frozen backbone still requires a full forward pass at each training step, with LoRA-GRL adding further computation from the parallel adapter path and domain-discriminator branch that full fine-tuning does not require.

On the measured GPU (Table 12), unmerged LoRA-GRL averaged 75.9 ± 4.2 ms per selected input, versus 38.5 ± 3.0 ms for Full-FT. After merging, LoRA-GRL averaged 38.6 ± 3.2 ms (35.0 inputs/s at batch size 8). This observed forward-pass timing is similar to Full-FT in this benchmark. Merging removes the additional low-rank operations from the forward graph; it does not establish zero end-to-end clinical processing overhead or statistical equivalence of latency.

Absolute latency depends on hardware, software, numeric precision, and benchmark protocol. The CPU results (Table 13) are an indicative hardware reference and show greater variation across methods. We interpret the measured GPU values as forward-pass timings on this device rather than generalizable clinical processing times.

### 3.6. Qualitative Grad-CAM Analysis

**Figure 6:**
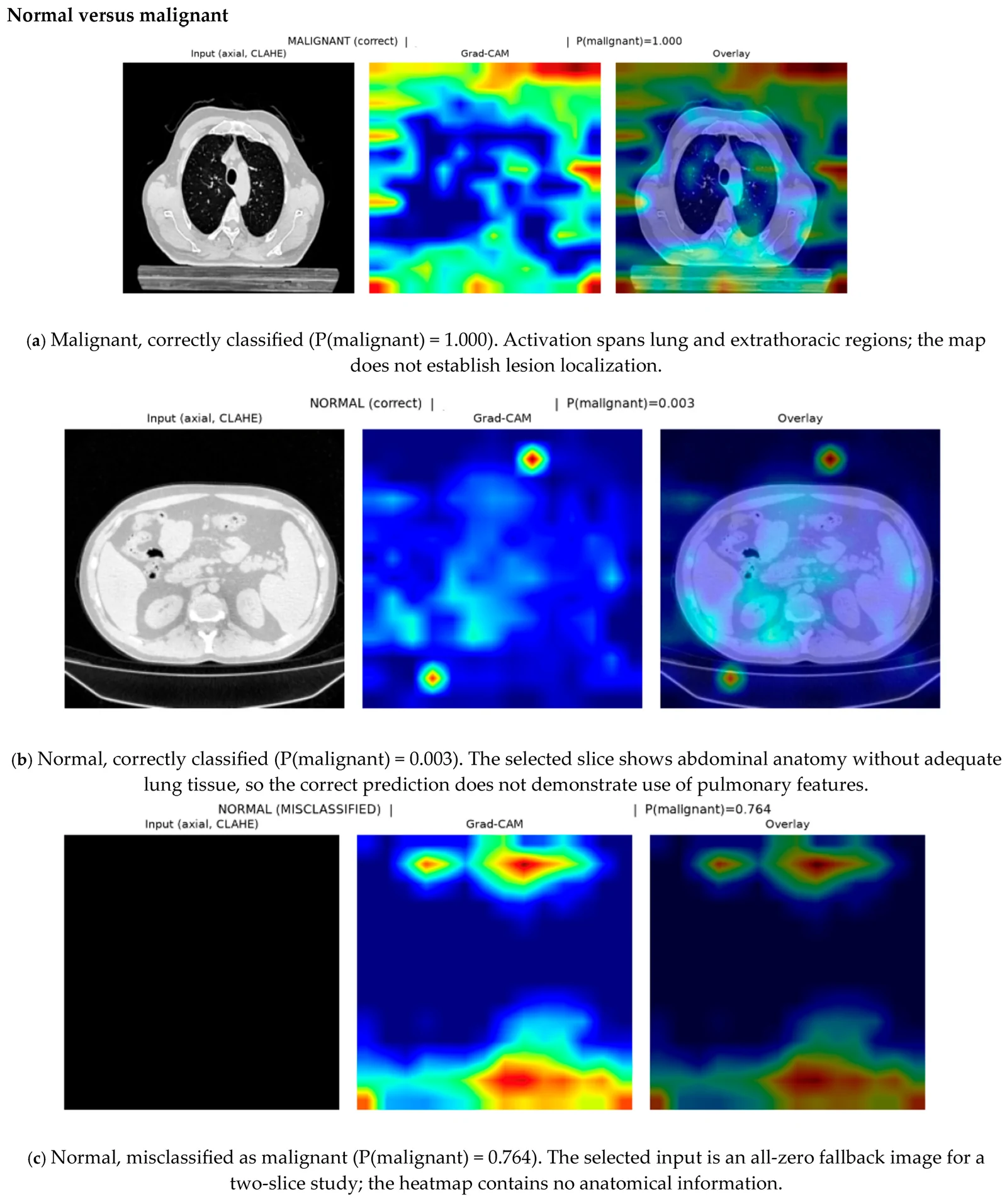

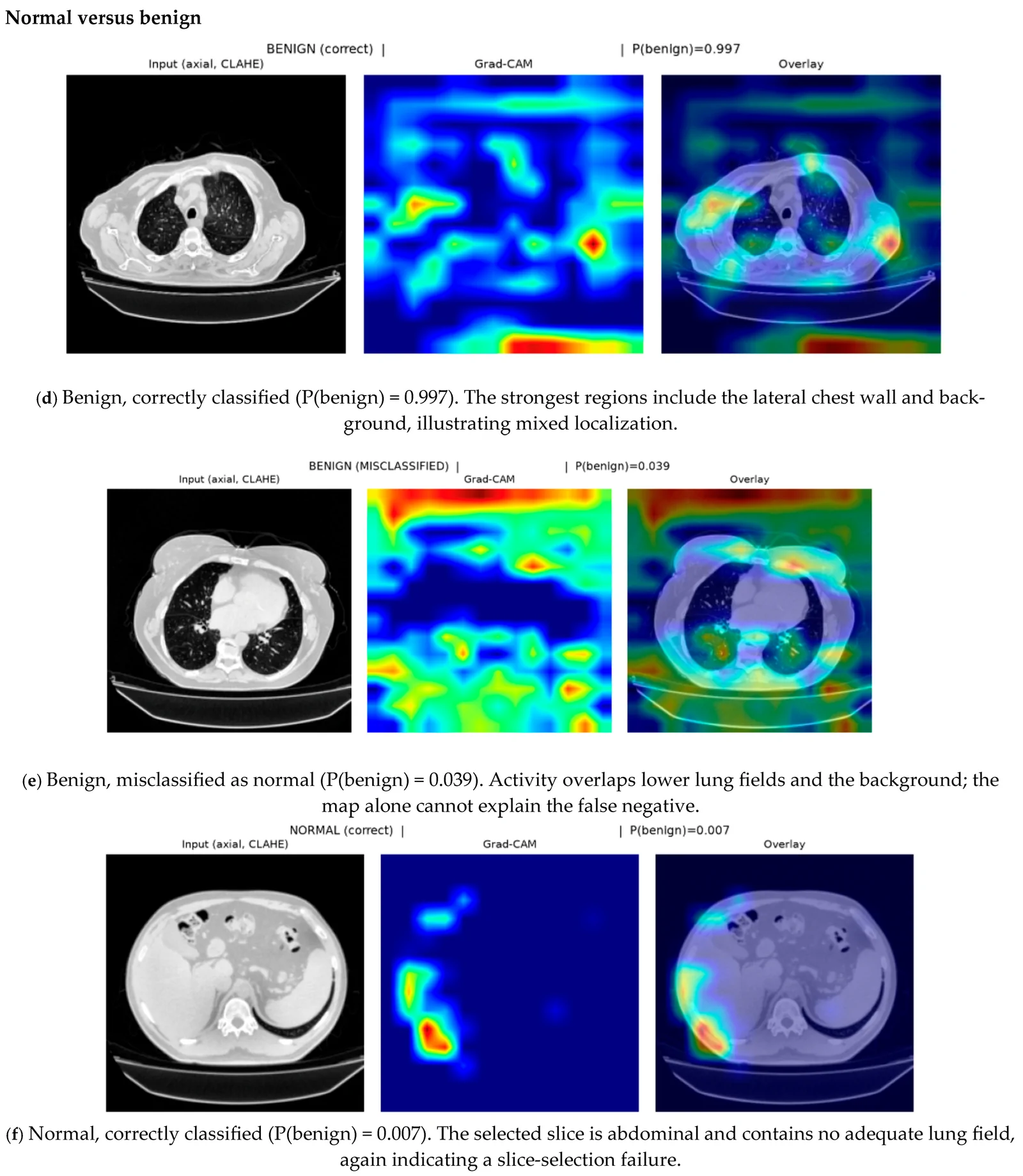
ViT-adapted Grad-CAM on selected held-out patients from separate single-split models. Panels (**a**–**c**) show the malignant task and panels (**d**–**f**) the benign task. Each image shows the selected axial input, heatmap, and overlay. The selection deliberately includes correct and incorrect predictions and input-quality failures.

To provide an exploratory visual assessment of the image regions associated with LoRA-GRL predictions, we applied a ViT-adapted Grad-CAM to held-out cases from separate patient-level splits of the 124-study development subset. Because the classification head pools only the CLS token, gradients were computed with respect to patch-token activations in the second-to-last transformer block, averaged across the 196 patch locations, ReLU-weighted, reshaped to a 14 × 14 grid, and upsampled to 224 × 224 for overlay. Figure 6 shows six representative examples from single-split models trained for this analysis; these are not the five-fold models used for the primary performance estimates, and the displayed probabilities are not new performance estimates. Panels (a)–(c) show the Normal-versus-Malignant task and panels (d)–(f) the Normal-versus-Benign task, deliberately including correct predictions, misclassifications, and input-quality failures. These maps are exploratory visualizations rather than causal explanations or validated localization metrics.

## 4. Discussion

This study proposed LoRA-GRL, a parameter-efficient and domain-harmonized Vision Transformer framework for multi-center lung nodule classification. The main finding is that combining Low-Rank Adaptation with adversarial domain alignment can achieve performance comparable to full fine-tuning while reducing the number of trainable parameters by more than 96%. This finding is important because medical imaging datasets are often modest in size, institutionally heterogeneous, and difficult to centralize [15]. Under such conditions, full-network fine-tuning may increase the risk of overfitting to center-specific image characteristics, whereas parameter-efficient adaptation can preserve the general visual representations learned during large-scale pretraining while allowing task-specific specialization [34].

When the pre-specified comparisons were evaluated (LoRA-GRL versus plain LoRA and versus Full-FT on both tasks), the AUC differences were not statistically distinguishable after Bonferroni correction, and the paired confidence intervals included zero. These findings indicate that LoRA-GRL achieves discrimination comparable to full fine-tuning and to the parameter-efficient baselines rather than outperforming them. Its distinguishing contribution is therefore efficiency, a 96.4% reduction in trainable parameters, with correspondingly smaller optimizer and storage footprints, together with the cross-center consistency conferred by the adversarial objective, rather than a measured accuracy advantage. This distinction is important because it suggests that the advantages of LoRA-GRL arise from efficient, domain-aligned adaptation rather than from greater model capacity.

The results indicate that model capacity alone was not the main determinant of performance. Full fine-tuning updated 21.865 million parameters and achieved strong discrimination, particularly for the Normal versus Benign task. However, LoRA-GRL reached a nearly identical AUC for this task, comparable to full fine-tuning for the Normal versus Malignant task while updating only 0.789 million parameters. This supports the growing evidence that parameter-efficient fine-tuning is well suited to medical imaging, where limited sample sizes and high acquisition variability make full optimization of large models less efficient [20,21]. Recent benchmarks have similarly shown that methods such as LoRA can perform competitively across medical image classification tasks while requiring substantially fewer trainable parameters than conventional fine-tuning [35,36]. In the specific context of lung nodule analysis, recent work on LoRA-adapted large vision models reported that low-rank adaptation can improve malignancy classification while reducing computational burden. The present study extends this line of evidence by showing that LoRA can also be integrated with explicit domain harmonization in a multi-institutional CT setting.

Measured inference latency clarifies the efficiency claim. Used as trained, with the low-rank branch active, LoRA-GRL was about twice as slow as full fine-tuning because the parallel adapter matrix multiplications are not fused with the frozen layers; once the adapters were merged into the backbone weights, the standard deployment step, its latency became statistically indistinguishable from full fine-tuning while retaining the parameter, memory, and storage advantages. Deployment should therefore use the merged model, for which the efficiency gains carry no inference-time penalty.

A key distinction between the proposed framework and previous LoRA-based medical imaging applications [37] is the explicit treatment of domain shift [14,15]. Although LoRA reduces the number of trainable parameters, it does not by itself guarantee that the learned representation will be invariant to institutional differences. This limitation is particularly relevant in thoracic CT, where scanner manufacturer, reconstruction kernel, slice thickness, dose protocol, and local preprocessing pipelines can introduce systematic intensity and texture differences [15]. The centers also differ markedly in the number of axial slices exported per study, from curated key-slice sets to near-complete stacks, a further, previously unquantified source of cross-center heterogeneity that the domain-adversarial component must accommodate. These differences may be exploited by deep learning models as shortcut features, resulting in high internal performance but unstable external generalization. The cross-center results in this study directly address this issue. LoRA-GRL achieved the highest mean AUC across centers for both diagnostic tasks and produced the smallest cross-center AUC range for malignant nodule classification. This indicates that the GRL component successfully reduced the dependence of the learned features on center-specific imaging characteristics. Nevertheless, because center identity is correlated with the class distribution in this cohort (Al-Makassed benign-dominant, Augusta Victoria malignant-dominant), the domain-adversarial objective cannot be cleanly separated from label-correlated anatomical variation; the improved cross-center consistency should therefore be read as evidence that the GRL reduces center-associated variance rather than as proof that it isolates scanner- and protocol-specific signal from disease-correlated anatomy. Disentangling the two would require a center-balanced or label-stratified design, which we identify as part of the planned leave-one-center-out and external-validation work.

The improvement obtained by adding the Gradient Reversal Layer is consistent with the broader domain adaptation literature. Domain-adversarial learning encourages the feature extractor to retain task-discriminative information while suppressing domain-identifying signals [16]. In medical imaging, similar approaches have been used to reduce scanner- and site-related variability in histopathology, retinal imaging, and MRI [17,19]. However, most prior domain-adaptation frameworks rely on full-network optimization. The present study demonstrates that adversarial domain learning remains effective even when adaptation is restricted to low-rank updates within the attention and feed-forward (MLP) projections of a frozen Vision Transformer [38]. This is scientifically relevant because it indicates that the low-rank adaptation space may be sufficiently expressive not only for disease discrimination but also for suppressing domain-specific variation.

The ablation study further clarifies the contribution of each component. Standard LoRA provided a strong parameter-efficient baseline, but adding GRL improved AUC, accuracy, weighted F1-score, and sensitivity for malignant nodule classification without increasing the trainable parameter count. The subsequent addition of Frobenius-norm regularization further improved performance, indicating that constraining the magnitude of adapter updates reduced over-specialization to the training-center distribution. Finally, CLAHE-based contrast enhancement produced the highest overall performance, indicating that local contrast normalization remained beneficial even in a transformer-based framework. These incremental gains show that the final performance was not attributable to a single component but resulted from the combined effect of domain alignment, regularized low-rank adaptation, and contrast-enhanced input representation [14,15].

The Precision, MCC, and PR-AUC metrics reinforce this reading and are reported without selecting favorable cases. On the Normal-versus-Malignant task, LoRA-GRL remained competitive across all metrics, whereas on the Normal-versus-Benign task it was among the weaker methods on the threshold-independent metrics, with the lowest PR-AUC and one of the lowest MCC values, consistent with its lower benign-task sensitivity. This task-dependent behavior underscores that the framework’s value is efficiency and cross-center robustness rather than uniform accuracy superiority.

The sensitivity improvement in the Normal versus Malignant task is particularly relevant from a clinical perspective. LoRA-GRL increased sensitivity compared with full fine-tuning while maintaining the same specificity. In lung cancer screening, false negatives are clinically more concerning than false positives because missed malignant nodules may delay diagnosis and treatment. Therefore, a method that improves malignant nodule detection without increasing false-positive classifications has potential clinical value. The ROC analysis further supports this interpretation, as LoRA-GRL showed strong discrimination at low false-positive rates, especially in the malignant task.

The clinically motivated task decomposition also contributed to the interpretability of the framework. Instead of forcing the model to learn a simultaneous three-class decision boundary, the study separated the diagnostic process into two binary tasks: Normal versus Benign and Normal versus Malignant. This formulation is closer to the sequential logic of radiological assessment, where the first decision concerns the presence of an abnormal finding and the second concerns the likelihood of malignancy. Methodologically, this decomposition reduces class heterogeneity and allows each classifier to focus on a more specific diagnostic contrast. This may partly explain the strong performance observed across both tasks, particularly in malignant nodule detection.

Compared with recent lung nodule classification studies, the main contribution of this work is not simply achieving a high AUC but achieving high and stable performance under multi-center heterogeneity with a small trainable parameter budget. Many recent studies have reported strong performance using CNNs, ViTs, self-supervised models, or hybrid architectures, but fewer have directly evaluated the joint problem of parameter efficiency and cross-center robustness (Table 10). Similarly, recent PEFT studies in medical imaging have emphasized computational efficiency, while domain adaptation studies have emphasized robustness. LoRA-GRL bridges these two directions by showing that a parameter-efficient ViT can be adapted in a domain-adversarial manner without requiring full-network fine-tuning.

To move interpretability from discussion to demonstration, we applied a Vision-Transformer-adapted Grad-CAM to held-out cases. Localization was inconsistent: some maps concentrated plausibly on lung or lesion tissue, while others were dominated by background or extrathoracic structures, and one “normal” case was classified correctly only because the automatically selected axial slice was extrathoracic. Rather than weakening the study, these observations provide direct visual support for its acknowledged limitations, particularly the annotation-free slice-selection heuristic, and reinforce both the proof-of-concept framing and the need for nodule-centered cropping and improved slice selection in future work.

### Limitations

Several limitations should be acknowledged. The study lacks external validation, as all data were collected from three centers within a single country; while the model exhibited a narrow cross-center performance range within this cohort, whether this invariance extends to populations and imaging protocols outside Palestine remains untested. It also did not include a Benign-versus-Malignant classification task; given that the Normal-versus-Malignant AUC (0.9869) exceeded the Normal-versus-Benign AUC (0.9735) in this work, this more challenging distinction is a natural priority for future investigation. From a methodological standpoint, the slice selection method, which picks the axial slice with the highest soft-tissue content, may miss nodules located outside the selected slice, potentially underestimating true performance, although a check against radiologist-recorded best-view slice ranges found the automatically selected slice within the recorded nodule range in 83% of benign and 93% of malignant evaluable cases (median offset for misses, 53 slices); in addition, a full hyperparameter search over the LoRA rank and GRL coefficient was not undertaken given the modest multi-center sample size, although a limited sensitivity analysis for these two parameters is now reported in Section 3.3 (Table 7). Finally, an explainability method tailored to the LoRA-GRL architecture and its 2.5D multi-planar input could be designed. These aspects will be addressed in future work.

## 5. Conclusions

This study introduced LoRA-GRL, a parameter-efficient framework that integrates Low-Rank Adaptation with adversarial domain harmonization for multi-center lung nodule classification using chest CT images. By combining lightweight low-rank adapters with a Gradient Reversal Layer in a frozen Vision Transformer, the proposed framework showed encouraging evidence of reducing inter-center domain shift while substantially lowering storage and memory requirements, although confirmation in external cohorts is required.

Evaluation on a multi-institutional dataset demonstrated that LoRA-GRL achieved classification performance comparable to full fine-tuning while reducing the number of trainable parameters by more than 96%. In addition, the framework consistently improved cross-center robustness, suggesting that parameter-efficient adaptation and adversarial domain alignment can jointly enhance the generalizability of Vision Transformer models across heterogeneous clinical environments. When the low-rank adapters are merged for deployment, this efficiency is achieved without any inference-time penalty relative to full fine-tuning, and an exploratory Grad-CAM analysis provided preliminary, qualitative evidence of nodule-relevant attention alongside clear failure modes to be addressed in future work. Future work should include external validation in larger, multi-national cohorts, extension to full 3D volumetric analysis, incorporation of a Benign vs. Malignant classification task, and prospective clinical testing to establish real-world utility.

## Figures and Tables

**Table 1:** (**a**) CT scanner, acquisition parameters, and reconstruction protocols by participating center. (**b**) CT volumes and axial slices by center in the 264-patient analysis cohort.

(**a**)				
**Center**	**Protocol**	**Scanner**	**Acquisition Parameters**	**Reconstruction Details**
Al-Makassed	HRCT chest	Philips Incisive 128-slice	100 kVp; 64 × 0.625 mm collimation; pitch 0.60; rotation 0.5 s; DoseRight, 3D dose modulation; inspiration breath-hold	Mediastinal: 3/1.5 mm, iDose 4, filter B, 768 × 768, FOV 350 mm, WC 40/WW 400; Lung: 1/0.5 mm, iDose 3, filter A, enhancement 1, 1024 × 1024, FOV 350 mm, WC −600/WW 1600; Coronal MPR: 0.9–1.0/0.5 mm, iDose 4, YD, AIP
Al-Makassed	Non-contrast chest	Philips Incisive 128-slice	120 kVp; 64 × 0.625 mm collimation; pitch 0.60; rotation 0.5 s; standard resolution; DoseRight, 3D dose modulation	Mediastinal: 3/1.5 mm, iDose 4, filter B, 512 × 512, FOV 350 mm, WC 40/WW 400; Lung: 1/0.5 mm, iDose 3, filter YA, 512 × 512, WC −600/WW 1600; Coronal lung: 1/0.5 mm, iDose 4, YA, AIP; Bone: 1/0.5 mm, iDose 4, YA, 512 × 512, WC 500/WW 1600
Al-Makassed	Chest/abdomen	Philips Incisive 128-slice	120 kVp; 64 × 0.625 mm collimation; pitch 1.0; rotation 0.75 s; DoseRight, 3D dose modulation; inspiration breath-hold	Unenhanced axial: 4/2 mm, iDose 4, B, 512 × 512, abdominal WC 60/WW 360; Contrast-enhanced axial: 4/2 mm, iDose 4, B, 512 × 512; Sagittal/coronal MPR: 3/1.5 mm, iDose 4, B, AIP; Lung: 2/1 mm, iDose 4, YA, enhancement 1, 512 × 512, WC −600/WW 1600; Coronal reformations: 2/1 mm, iDose 4, B, AIP
St. Joseph	Non-contrast chest	Philips Incisive multidetector	120 kVp; DRI 25; 64 × 0.625 mm collimation; pitch 1.0; rotation 0.4 s; standard resolution; 3D dose modulation	Mediastinal: 3/3 mm, iDose 3, B, AIP, 512 × 512, WC 40/WW 400; Sagittal/coronal MPR: 3/3 mm, iDose 3, B, AIP; Lung HR: 1/0.5 mm, iDose 5, YB, AIP, 512 × 512, WC −600/WW 1600
St. Joseph	Chest–abdomen	Philips Incisive multidetector	120 kVp; 64 × 0.625 mm collimation; pitch 1.0; rotation 0.5 s; standard resolution; DoseRight, 3D dose modulation	Axial abdomen: 3/1.5 mm, iDose 3, B, AIP, 512 × 512, WC −15/WW 496; Lung HR: 1/1 mm, iDose 3, YA, AIP, 512 × 512, WC −600/WW 1600; Sagittal/coronal MPR: 3/1.5 mm
(**b**)				
**Center**		**CT Volumes (Patients)**	**Total Axial Slices**	**Mean Slices/Patient**
Al-Makassed		87	7508	86.3
Augusta Victoria		87	5474	62.9
St Joseph		90	7482	83.1
Total		264	20,464	77.5

**Table 2:** Precision, MCC, and PR-AUC: normal versus malignant (development subset).

Method	Precision	MCC	PR-AUC	AUC	Sens.	WF1
No-FT	0.8491	0.6699	0.9443	0.8994	0.900	0.842
Partial-FT	0.7800	0.4467	0.8864	0.8164	0.780	0.735
Full-FT	0.9388	0.8251	0.9674	0.9527	0.920	0.916
LoRA	0.8846	0.7468	0.9382	0.9245	0.920	0.879
LoRA-GRL *	0.9130	0.7077	0.9573	0.9206	0.840	0.857
BitFit	0.8958	0.7021	0.9403	0.9145	0.860	0.856
Adapter	0.9000	0.7485	0.9665	0.9424	0.900	0.879
VPT	0.8113	0.5674	0.9258	0.8730	0.860	0.793
DANN (full + GRL)	0.8936	0.6799	0.9376	0.9170	0.840	0.844

* Proposed method.

**Table 3:** Precision, MCC, and PR-AUC: normal versus benign (development subset).

Method	Precision	MCC	PR-AUC	AUC	Sens.	WF1
No-FT	0.7500	0.5356	0.9317	0.9091	0.878	0.766
Partial-FT	0.8409	0.6989	0.9354	0.9120	0.902	0.850
Full-FT	0.8571	0.6986	0.9304	0.9076	0.878	0.851
LoRA	0.7826	0.5893	0.9117	0.8928	0.878	0.795
LoRA-GRL *	0.7907	0.5607	0.8772	0.8729	0.829	0.783
BitFit	0.8537	0.6718	0.9363	0.9187	0.854	0.838
Adapter	0.8182	0.6435	0.9494	0.9424	0.878	0.823
VPT	0.8043	0.6454	0.9304	0.9154	0.902	0.822
DANN (full + GRL)	0.8667	0.7834	0.9586	0.9490	0.951	0.891

* Proposed method.

**Table 4:** Comparison of Classification Performance among Methods.

Method	AUC	WF1	ACC	Sens.	Spec.	Params (M)	S/EP
Task 1—Benign (*N* = 162 Patients)							
No-FT	0.9520	0.9019	0.9012	0.8750	0.9556	0.199	0.8
Partial-FT	0.9596	0.9141	0.9136	0.8958	0.9556	5.523	0.9
Full-FT	0.9747	0.9384	0.9383	0.9375	0.9556	21.865	0.8
LoRA	0.9624	0.9262	0.9259	0.9167	0.9444	0.789	1.1
LoRA-GRL	0.9735	0.9141	0.9136	0.8958	0.9667	0.789	1.1
Task 2—Malignant (*N* = 168 Patients)							
No-FT	0.9638	0.9055	0.9048	0.8824	0.9697	0.199	0.9
Partial-FT	0.9489	0.8939	0.8929	0.8627	0.9394	5.523	0.9
Full-FT	0.9863	0.9175	0.9167	0.8824	0.9697	21.865	0.8
LoRA	0.9715	0.9175	0.9167	0.8824	0.9697	0.789	1.1
LoRA-GRL	0.9869	0.9286	0.9286	0.9412	0.9697	0.789	1.2

Sens. = Sensitivity (disease class recall). Spec. = Specificity (Normal class recall). s/ep = mean seconds per training epoch on RTX 4070 Ti. Params (M) = millions of trainable parameters.

**Table 5:** Per-Centre ROC-AUC.

Method	Almakassed	Augusta	St. Joseph	Mean
Task 1—Benign				
No-FT	0.9686	0.9140	0.9830	0.9552
Partial-FT	0.9686	0.9517	0.9877	0.9693
Full-FT	0.9857	0.9494	0.9900	0.9750
LoRA	0.9643	0.9418	0.9855	0.9639
LoRA-GRL	0.9621	0.9879	0.9922	0.9807
Task 2—Malignant				
No-FT	0.9670	0.9760	0.9895	0.9775
Partial-FT	0.9593	0.9624	0.9858	0.9692
Full-FT	0.9835	0.9829	0.9920	0.9861
LoRA	0.9177	0.9937	0.9834	0.9649
LoRA-GRL	0.9945	0.9934	0.9941	0.9940

Mean = arithmetic average of the three center AUC values. Cross-center range (max–min) for LoRA-GRL on Normal vs. Malignant = 0.0011, the smallest of any method.

**Table 6:** Ablation Study—Normal vs. Malignant Task.

Configuration	AUC	WF1	ACC	Sens.	Params (M)
Full-FT [upper reference]	0.9863	0.9175	0.9167	0.8824	21.865
LoRA (no GRL, no L_reg)	0.9715	0.9175	0.9167	0.8824	0.789
+GRL (no L_reg)	0.9792	0.9203	0.9226	0.9020	0.789
+GRL + L_reg (no CLAHE)	0.9821	0.9241	0.9247	0.9216	0.789
+GRL + L_reg + CLAHE [LoRA-GRL]	0.9869	0.9286	0.9286	0.9412	0.789

Components added incrementally. Full Fine-Tuning is the non-PEFT upper reference. The highlighted row is the proposed LoRA-GRL. L_reg = Frobenius-norm regularization on LoRA adapters. All LoRA variants use identical parameter counts.

**Table 7:** Effect of LoRA rank and GRL coefficient (Normal vs. Malignant, local subset).

Varying	*r*	*λ*_d_	AUC (Pooled) ± SD	AUC 95% CI	WF1	Sens.	Spec.	PR-AUC	MCC
LoRA rank (*λ*_d_ = 0.5)	4	0.5	0.848 ± 0.030	[0.762, 0.922]	0.749	0.760	0.723	0.906	0.481
LoRA rank (*λ*_d_ = 0.5)	8	0.5	0.913 ± 0.039 *	[0.846, 0.968]	0.868	0.860	0.867	0.945	0.730
LoRA rank (*λ*_d_ = 0.5)	16	0.5	0.897 ± 0.026	[0.823, 0.959]	0.821	0.800	0.838	0.923	0.637
GRL coefficient (*r* = 8)	8	0.1	0.947 ± 0.047	[0.894, 0.987]	0.891	0.920	0.833	0.961	0.773
GRL coefficient (*r* = 8)	8	0.5	0.913 ± 0.039 *	[0.846, 0.968]	0.868	0.860	0.867	0.945	0.730
GRL coefficient (*r* = 8)	8	1.0	0.876 ± 0.071	[0.796, 0.943]	0.797	0.780	0.800	0.919	0.588

* Reference LoRA-GRL configuration. Trainable adapter parameters: *r* = 4 → 0.44 M, *r* = 8 → 0.74 M, *r* = 16 → 1.33 M. Pooled AUC = macro ROC-AUC over concatenated held-out folds; SD = standard deviation of the five per-fold AUCs; Sens. = pooled recall of the malignant class; Spec. = mean recall of the normal class across folds; CI = patient-level bootstrap 95% confidence interval.

**Table 8:** Competing methods under an identical protocol (Normal vs. Malignant, patient-level 5-fold CV; development subset).

Method	Trainable Params (M)	AUC (Pooled) ± SD	AUC 95% CI	WF1	Sens.	Spec.	PR-AUC	MCC
BitFit	0.200	0.9145 ± 0.0185	[0.846, 0.968]	0.8561	0.8600	0.8333	0.9403	0.7021
Adapter	1.339	0.9424 ± 0.0296	[0.891, 0.983]	0.8795	0.9000	0.8429	0.9665	0.7485
VPT	0.153	0.8730 ± 0.0628	[0.790, 0.940]	0.7933	0.8600	0.6940	0.9258	0.5674
DANN (full + GRL)	21.814	0.9170 ± 0.0421	[0.844, 0.974]	0.8444	0.8400	0.8333	0.9376	0.6799
Full-FT	21.814	0.9527 ± 0.0245	[0.904, 0.990]	0.9159	0.9200	0.9000	0.9674	0.8251
LoRA	0.739	0.9245 ± 0.0377	[0.852, 0.979]	0.8788	0.9200	0.8000	0.9382	0.7468
LoRA-GRL *	0.739	0.9206 ± 0.0517	[0.853, 0.974]	0.8565	0.8400	0.8667	0.9573	0.7077

* Proposed method. All rows: identical data, patient-level splits, preprocessing, augmentation, class balancing and early-stopping rule. Pooled AUC = macro ROC-AUC over the concatenated held-out folds; SD = standard deviation of the five per-fold AUCs; CI = patient-level bootstrap 95% confidence interval.

**Table 9:** (**a**) Paired pooled-AUC differences against LoRA-GRL (patient-level bootstrap, same patients; development subset). (**b**) Pairwise ROC-AUC comparisons: normal versus malignant (development subset). (**c**) Pairwise ROC-AUC comparisons: normal versus benign (development subset).

(**a**)						
**Comparison**		**ΔAUC**	**95% CI**		**Bootstrap *p***	
LoRA-GRL − LoRA		−0.0039	[−0.0561, +0.0516]		0.874 (n.s.)	
LoRA-GRL − Full-FT		−0.0321	[−0.0873, +0.0116]		0.182 (n.s.)	
LoRA-GRL − Adapter		−0.0218	[−0.0682, +0.0176]		0.314 (n.s.)	
LoRA-GRL − DANN (full + GRL)		+0.0036	[−0.0506, +0.0542]		0.873 (n.s.)	
LoRA-GRL − BitFit		+0.0061	[−0.0535, +0.0612]		0.812 (n.s.)	
LoRA-GRL − VPT		+0.0476	[−0.0230, +0.1225]		0.192 (n.s.)	
(**b**)						
**Comparison**	**AUC, LoRA-GRL (95% CI)**	**AUC, Rival (95% CI)**	**ΔAUC**	**95% CI (ΔAUC)**	**Perm.** ***p** ***(raw)**	***p*** **(Bonf. ×4)**
LoRA-GRL vs. LoRA	0.9206 [0.853, 0.974]	0.9245 [0.852, 0.979]	−0.0039	[−0.0558, +0.0503]	0.924	1.000
LoRA-GRL vs. Full-FT	0.9206 [0.853, 0.974]	0.9527 [0.904, 0.990]	−0.0321	[−0.0882, +0.0128]	0.242	0.967
(**c**)						
**Comparison**	**AUC, LoRA-GRL (95% CI)**	**AUC, Rival (95% CI)**	**ΔAUC**	**95% CI (ΔAUC)**	**Perm.** ***p*** **(raw)**	***p*** **(Bonf. ×4)**
LoRA-GRL vs. LoRA	0.8729 [0.779, 0.949]	0.8928 [0.814, 0.956]	−0.0200	[−0.0868, +0.0382]	0.538	1.000
LoRA-GRL vs. Full-FT	0.8729 [0.779, 0.949]	0.9076 [0.828, 0.970]	−0.0347	[−0.1001, +0.0314]	0.272	1.000

Paired resampling draws the same patient indices for both methods (the fold split is identical across methods). A confidence interval that includes 0 indicates that the pooled-AUC difference is not distinguishable from chance at this sample size. n.s. stands for not significant.

**Table 10:** Comparison with recent lung nodule classification studies.

Study	Method	Dataset	Domain Adaptation	Parameter Efficiency	Reported AUC
This work	LoRA + GRL	3 centers, Palestine	Yes (GRL)	Yes (0.789 M)	0.9735–0.9869
[26]	LoRA (PEFT) on SwinV2-b	LIDC	No	Yes (1.5 M)	0.970
[32]	Full FT, dual-branch DCSwinB	LUNA16-K	No	No	0.94
[33]	Full FT, hybrid C-Swin	IQ-OTH/NCCD	No	No	0.93–0.97

**Table 11:** Overlap between the automatically selected slice and the radiologists’ recorded nodule range.

Case Set	Benign Within Range	Malignant Within Range	Overall
All matched cases	72/96 (75.0%)	92/102 (90.2%)	164/198 (82.8%)
Pipeline input contains annotated region	72/81 (88.9%)	92/97 (94.8%)	164/178 (92.1%)

**Table 12:** GPU model forward-pass latency (NVIDIA T550, 4 GB; batch size 1, 300 timed passes).

Method	Latency b = 1, ms (Mean ± SD)	Median	p95	Throughput b = 8 (img/s)
No-FT	40.92 ± 13.71	38.56	43.72	34.8
Partial-FT	40.72 ± 12.33	39.04	42.10	35.0
BitFit	41.01 ± 22.29	38.93	41.59	34.8
Adapter	44.89 ± 15.38	42.41	48.61	33.1
VPT	39.17 ± 2.15	38.94	40.09	35.1
Full-FT/DANN	38.50 ± 2.99	38.20	38.96	35.2
LoRA (unmerged)	75.78 ± 3.16	75.16	77.72	18.4
LoRA (merged)	40.17 ± 6.60	38.97	44.62	34.9
LoRA-GRL (unmerged)	75.92 ± 4.16	75.44	77.49	18.3
LoRA-GRL (merged)	38.62 ± 3.21	38.21	39.67	35.0

**Table 13:** CPU model forward-pass latency (hardware reference).

Method	Latency b = 1, ms (Mean ± SD)	Median	p95	Throughput b = 8 (img/s)
No-FT	90.50 ± 9.70	89.84	107.42	18.1
Partial-FT	135.48 ± 5.80	134.78	144.09	10.1
BitFit	244.39 ± 58.77	214.85	384.80	15.3
Adapter	149.02 ± 16.38	150.33	172.29	11.5
VPT	135.97 ± 7.82	135.34	147.43	12.3
Full-FT/DANN	149.79 ± 25.30	144.93	184.94	13.6
LoRA (unmerged)	233.43 ± 14.71	235.23	254.03	7.3
LoRA (merged)	116.26 ± 4.80	115.89	123.21	13.1
LoRA-GRL (unmerged)	226.16 ± 11.85	225.14	239.47	7.5
LoRA-GRL (merged)	119.86 ± 16.51	118.64	153.45	14.0

## Data Availability

Data are available from the corresponding author upon reasonable request. Data are not publicly available due to ethical and privacy restrictions, as they consist of fully anonymized patient CT images obtained under Institutional Review Board approval that does not permit unrestricted public data sharing.

## Abbreviations

The following abbreviations are used in this manuscript:

LoRA-GRL	Low-Rank Adaptation with Gradient Reversal Layer
CT	computed tomography
LoRA	Low-Rank Adaptation
GRL	Gradient Reversal Layer
ViT-Small	Vision Transformer Small
AUC	Area Under the Receiver Operating Characteristic Curve
CAD	computer-aided diagnosis
CNNs	convolutional neural networks
ViTs	Vision Transformers
DANN	Domain-Adversarial Neural Network
MRI	Magnetic Resonance Imaging
PEFT	parameter-efficient fine-tuning
DICOM	Digital Imaging and Communications in Medicine
Lung-RADS	Lung Imaging Reporting and Data System
ACR	American College of Radiology
HU	Hounsfield Unit
CLAHE	Contrast-Limited Adaptive Histogram Equalization
Q	query
K	key
V	value
O	output
MLP	Multilayer Perceptron
ReLU	Rectified Linear Unit
LayerNorm	Layer Normalization
BCE	Binary Cross-Entropy
CE	Cross-Entropy
EMA	Exponential Moving Average
cuBLAS	CUDA Basic Linear Algebra Subprograms
WF1	Weighted F1-Score
ACC	Accuracy
ROC	Receiver Operating Characteristic
s/ep	seconds per epoch
